# Mathematical modeling of HBV–HDV transmission with superinfection: The role of vaccination and liver transplantation in disease dynamics

**DOI:** 10.1371/journal.pone.0356725

**Published:** 2026-09-09

**Authors:** Dipo Aldila, Nur Syam Rahman, Anymore Majere, Fatmawati

**Affiliations:** 1 Department of Mathematics, Faculty of Mathematics and Natural Sciences, Universitas Indonesia, Depok, Indonesia; 2 Innovative Mathematics and Predictive Analytics for Complex System and Technology Laboratory (IMPACT Lab), Universitas Indonesia, Depok, Indonesia; 3 Department of Mathematics, Faculty of Science and Technology, Universitas Airlangga, Surabaya, Indonesia; Japan Institute for Health Security, JAPAN

## Abstract

Hepatitis D virus (HDV) infection can substantially worsen the clinical outcomes of individuals infected with hepatitis B virus (HBV), making the prevention of HBV infection and the management of severe HBV–HDV cases important public-health priorities. This study introduces a novel mathematical model for Hepatitis B virus (HBV) and D virus (HDV) transmission, incorporating vaccination and liver transplantation as disease control strategies. In contrast to models that allow direct HDV infection or general coinfection pathways, the proposed model assumes that HDV occurs only through superinfection among individuals already infected with HBV. The model is constructed as a system of ordinary differential equations, where the human population is divided into susceptible, vaccinated, HBV-infected, recovered, chronic, and HDV-infected compartments. Model analysis reveals the existence and stability of the disease-free equilibrium, which exists and is asymptotically stable if the basic reproduction number (${ℛ}_{0}$) is less than one, and becomes unstable if it exceeds one. Two types of endemic equilibria are identified when ${ℛ}_{0}>1$, corresponding to the absence and presence of HDV infection. Continuation analysis using *MatCont* reveals the existence of two branching points that govern the transition of stability between these equilibria, highlighting the conditions under which HDV emerges and persists. Global sensitivity analysis using Partial Rank Correlation Coefficient combined with Latin Hypercube Sampling indicates that vaccination coverage and vaccine efficacy play a dominant role in reducing ${ℛ}_{0}$. Although liver transplantation does not affect the magnitude of ${ℛ}_{0}$, it significantly influences the disease dynamics by reducing the outbreak size and delaying the timing of HBV and HDV outbreaks. These findings indicate that vaccination is essential for reducing transmission, whereas liver transplantation remains important for mitigating severe disease burden; thus, coordinated use of preventive and clinical interventions is needed for effective HBV–HDV control.

## 1. Introduction

Hepatitis B virus (HBV) remains a major global public health concern, particularly in many low- and middle-income countries where vaccination coverage and access to healthcare services remain suboptimal. In addition, disease progression in HBV-infected individuals is often complicated by the possibility of coinfection with other diseases, including Hepatitis D virus (HDV), which significantly worsens clinical outcomes. Based on reports from the World Health Organization (WHO) [1], it is estimated that 254 million people were living with chronic HBV infection in 2022, with more than 1.2 million new cases annually. Of greater concern, HBV resulted in approximately 1.1 million deaths, mostly due to cirrhosis and hepatocellular carcinoma.

HBV is a liver infection caused by the hepatitis B virus. Individuals infected with HBV may experience either acute or chronic infection. On the other hand, HDV is a defective virus that requires the presence of HBV to replicate. Hence, HDV infection typically occurs either as superinfection or [2–4]. Epidemiological evidence suggests that superinfection, where HDV infects individuals who are already chronically infected with HBV, is more likely to lead to severe liver disease and may result in death. Understanding the interaction between HBV and HDV transmission is therefore essential for designing optimal strategies to control the spread of the disease [3,5–7].

Mathematical models have been widely used to understand the transmission of infectious diseases, such as dengue [8–10], malaria [11–14], tuberculosis [15–17], COVID-19 [18–21], HBV or HDV [22–25], and other communicable diseases [26,27]. The basic idea in these studies is to assume that the health status and types of interventions can be used to categorize the total human population into several distinct compartments. The transmission process and interactions between compartments are typically described using a transmission diagram, from which the mathematical model is derived. The basic reproduction number is commonly calculated in most epidemic modeling studies, where the results generally indicate that the disease has the potential to be eliminated if the basic reproduction number is less than one, and will persist if it is greater than one. In addition, multiple endemic equilibria or more complex dynamic behaviors may arise in more sophisticated models [9,27].

In the context of hepatitis transmission, several studies have been reported in the literature. To the best of our knowledge, the authors in [28] (1996) were among the first to introduce a specific compartmental epidemic model for HBV transmission. Their model incorporates waning vaccine-induced immunity and highlights the importance of booster doses. A simple SIR-type model was later introduced in [29] to investigate the impact of immune response at the scale of virus dynamics. Their analysis reveals the presence of forward bifurcation, indicating that the basic reproduction number of the virus serves as an essential threshold in determining the disease dynamics.

A number of recent HBV/HDV-related mathematical model studies have made strides well beyond the realms of simple susceptible-infected models to consider biologically more realistic processes and broader applicability. In 2023, authors in [30] formulated a detailed nonlinear model of HBV infection that included vaccination, therapy, migration, and screening, and found that prevention and screening are the key to reducing endemic rates. Moreover, the article by authors in [31] added to the HBV modeling by developing a model that considers passive immunity in infants, as well as screening and treatment of acute and chronic infections. Also, a biologically more realistic model was presented in the article by [32] that included temporary recovery, reinfection, failed vaccination, and waning immunity, which highlights that persistence of HBV cannot be characterized adequately when assuming permanent immunity. With the same spirit, [25] constructed a complex extended SIVRM model of the HBV dynamics in Indonesia, considering various modes of transmission, weakening immunity, and reactivation of HBV in recovered individuals. In this way, the model will be very applicable in achieving the objectives of WHO elimination strategy by 2030.

Another major development is the growing emphasis on qualitative analysis and control optimization. In 2023, authors in [33] examined acute and chronic HBV stages, thereby showing that reducing the reproduction number below unity may not always be sufficient for elimination. Authors in [34] then extended this control-oriented perspective by incorporating treatment failure and progression to severe liver outcomes such as cirrhosis and hepatocellular carcinoma. The combine sensitivity analysis with optimal control to show that multi-pronged strategies—condom use, vaccination, treatment, and behavioral change—outperform isolated interventions. In a similar manner, authors in [32] showed, through optimal control, that education, vaccination, and therapy are most effective when applied jointly rather than separately. Beyond mono-infection models, [35] studied HIV/HBV co-infection and showed through cost-effectiveness analysis that targeted combinations of HIV protection and HBV treatment may provide the most economically efficient strategy, while [36] advanced this line further by accounting for drug-induced hepatotoxicity and identifying integrated protection-and-treatment strategies as the most effective.

The other fascinating new trend is related to fractional-order dynamics and modeling of HBV/HDV interaction. A Caputo fractional HBV model with vaccine effects, acute and chronic carrier phases, and ANN-aided computational analysis was presented in 2024 by authors in [37]. They show how memory-dependent formulations reveal dynamics not captured by integer-order systems. A population-level HBV–HDV co-infection model incorporating awareness, vaccination, and treatment interventions was also proposed by Aja et al. [38]; however, it considers a general co-infection framework rather than explicitly modelling HDV transmission through superinfection among individuals already infected with HBV. In the case of HDV-related modeling, especially in superinfection models, there are not so many literature discuss this. Authors in [39] presented an early HBV–HDV kinetic model during anti-HDV therapy that explains the clinically observed rise of HBV when HDV declines, an interaction neglected in many earlier frameworks. Although, works by authors in [40] remains a useful foundational reference as it established the broader mathematical basis for HDV co-infection and super-infection dynamics. Collectively, these studies indicate a clear trend toward structurally detailed HBV models that better capture relapse, loss of protection, demographic pathways, and intervention heterogeneity, thus improving both epidemiological realism and public-health interpretability. Despite these advances, important aspects of intervention integration and co-infection mechanism insufficiently explored. Furthermore, population-level transmission models that explicitly formulate HDV acquisition solely through superinfection of individuals already infected with HBV remain scarce.

From a public health perspective, many authors believe that vaccination remains the cornerstone of HBV prevention, aiming to reduce the transmission probability in the population [30,40–42]. Achieving herd immunity in the population is often considered the minimum target. In parallel, clinical interventions such as liver transplantation play an important role in managing advanced-stage disease and are expected to reduce the severity of symptoms in infected individuals [37,43–45]. Although many mathematical models have been developed to study HBV transmission, only a limited number of studies discuss, in a unified framework, two important aspects, namely the inclusion of vaccination and liver transplantation in a single model, and the role of superinfection between HBV and HDV. The combined impact of these interventions in the context of HBV–HDV superinfection dynamics has not been fully explored.

Motivated by these gaps, this study aims to develop a novel mathematical model of HBV transmission incorporating HDV superinfection, vaccination, and liver transplantation strategies. The model is formulated as a system of ordinary differential equations that captures key epidemiological compartments and transition mechanisms, including vaccination rate, liver transplantation rate, recovery rate and its probability of treatment failure, as well as disease-induced mortality due to HBV–HDV infection. We perform rigorous mathematical analysis to investigate the existence and stability of the equilibrium points, complemented by numerical continuation methods to explore the bifurcation behavior. In addition, global sensitivity analysis is conducted to identify the most influential parameters affecting the basic reproduction number and the overall model dynamics.

The layout of this paper is as follows. In Section 2, we introduce the model assumptions and the process of model construction. Mathematical analysis regarding the existence and stability of the equilibrium points is presented in Section 3, where the basic reproduction number is also derived. Section 4 is devoted to numerical experiments, including continuation analysis using *MatCont* to produce bifurcation diagrams, global sensitivity analysis using the Partial Rank Correlation Coefficient method, level set analysis of the basic reproduction number, and autonomous simulations to investigate the impact of vaccination and liver transplantation on the model dynamics. In the final section, we present our conclusions.

## 2. Model construction

In this section, we introduced our novel mathematical model which aims to describe the transmission dynamics of hepatitis B virus (HBV) which later can be progress into hepatitis D virus infection (HDV). Furthermore, we also incorporate two types of intervention. First, we incorporate the vaccination strategy as a prevention of primary infection from HBV. Second, we incorporate the role of liver transplantation as a clinical intervention. We begin by giving a description of the variables and parameters, formulating a set of biological and epidemiological assumptions, and finally carefully construct the dynamical model for each variables. These assumptions reflect the interactions between susceptible and infected individuals, as well as the clinical pathways associated with advanced liver disease.

We divide the total of human population, denoted by *N*(*t*), into six compartments, namely:

1. The susceptible individuals, denoted by *S*(*t*). This group consist of individuals who are susceptible to HBV infection and able to get vaccination interventions.
2. The vaccinated individuals, denoted by *V*(*t*). This compartment consist of group of individuals who are already get vaccinated, and have a partial protection to HBV infection.
3. The infected individuals by HBV, denoted by $I_{b}(t)$. Individuals in this compartment are able to spread HBV, for example through blood transfusion, sexual contact, or any other path of infection.
4. The partial recovered individuals by HBV caused by treatment, denoted by *R*(*t*). This individuals had a temporal immunity respect to HBV, but still able to get infected by HDV. This individuals need a liver transplantation to get fully recovered by HBV.
5. The infected individuals by HBV in cronic stage, caused by failure treatment, and denoted by *C*(*t*).
6. The infected individuals by HDV, denoted by $I_{d}(t)$.

The model variables and parameters are summarized in Tables 1 and 2.

**Table 1:** List of variables in the hepatitis transmission model.

Variable	Definition	Value	Unit
*S*	Susceptible population to hepatitis B	S(t)≥0	persons
*V*	Vaccinated population against hepatitis B	V(t)≥0	persons
$I_{b}$	Population initially infected with hepatitis B	$I_{b}(t)\geq 0$	persons
*R*	Temporarily recovered population (can relapse)	R(t)≥0	persons
*C*	Chronic population due to treatment resistance	C(t)≥0	persons
$I_{d}$	Population infected with hepatitis D	$I_{D}(t)\geq 0$	persons

**Table 2:** Description of model parameters of system (1).

Par.	Description	Values	Unit	Ref.
Λ	Natural recruitment rate from newborn	$\frac{10000}{71\times 365}$	persons/year	Assumption
μ	Natural death rate	$\frac{1}{71\times 365}$	1/year	Assumption
$\delta_{b}$	Death rate induced by HBV infection	0.002	1/year	[52]
$\delta_{d}$	Death rate induced by HDV infection	0.001	1/year	Assumption
$\beta_{b}$	Effective contact rate of HBV infection	$\frac{0.35}{10000}$	1/ (person· year)	Adaptation from [53]
$\beta_{d}$	Effective contact rate of HDV infection	$\frac{0.33}{10000}$	1/ (person· year)	Assumption
*u*_1_	Vaccination rate	0.01	1/year	Assumption
*u*_2_	Rate of liver transplantation for HBV individuals	0.1	1/year	Assumption
α	Rate of waning of vaccination efficacy	0.1	1/year	[54]
ξ	Effectiveness of vaccination to reduce infection of HBV	0.8	–	Assumption
γ	Rate of HBV recovery	0.32	1/year	[55]
*p*	Proportion of perfect recovery from HBV	0.5	–	Assumption

Before we proceed on the model construction, we first introduced the mathematical assumptions that necessary to support our epidemiological meaning of our model.

A1 The total population assumed to be closed. Hence, no migration considered in this article.
A2 Although perinatal HBV transmission is epidemiologically important in some settings, and vertical HDV transmission has been reported rarely [46], these routes are beyond the scope of the present population-level model. Hence, vertical transmission of HBV and HDV is not incorporated in the present model. Accordingly, all newborns are assumed to enter the susceptible compartment. This simplification is adopted to focus on horizontal HBV transmission and subsequent HDV superinfection among HBV-infected individuals.
A3 HBV is transmitted through exposure to infectious blood and body fluids, including unsafe injections and contaminated medical equipment, whereas HDV follows similar blood-borne routes and can superinfect individuals with established HBV infection [5,47].
A4 Vaccination is assumed to be available only against HBV and has a spesific efficacy [48,49].
A5 Infected individuals have to get a proper treatment to get recovered by HBV.
A6 Since chronic HDV infection may persist and sustained virological clearance remains difficult to achieve [50], HDV-infected individuals are assumed not to recover in the present model.
A7 We assume that HBV treatment may fail with a certain probability. Individuals for whom treatment fails remain infected and are assumed to progress to the chronic HBV compartment.
A8 We assume that HDV infection occurs exclusively through superinfection [51]; that is, an individual with a pre-existing HBV infection subsequently acquires HDV. Therefore, HBV–HDV coinfection, in which both viruses are acquired simultaneously, is not considered in the present study.

**Fig 1:**
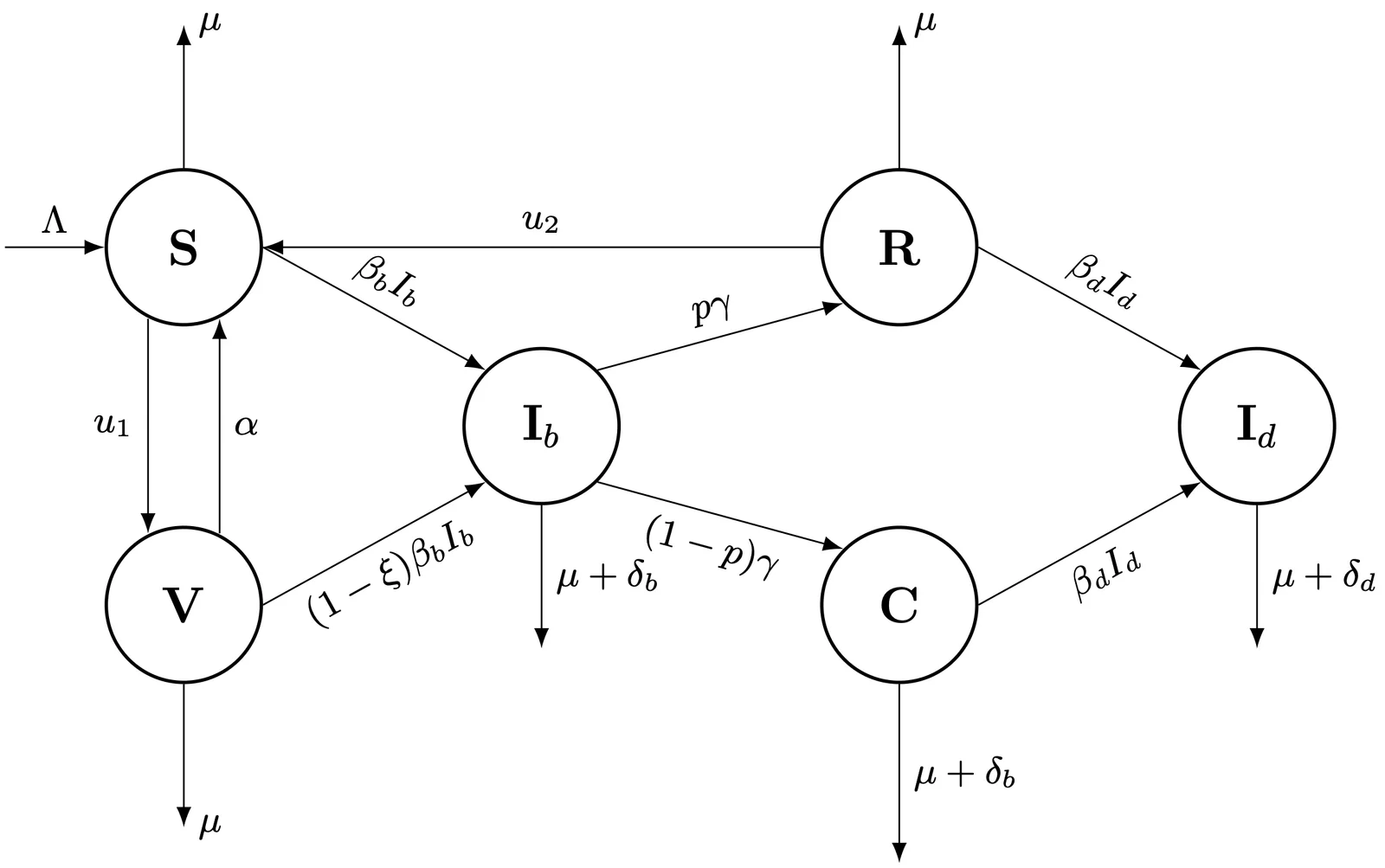
Schematic Diagram of the Model in (1) This transmission diagram illustrates the interactions among HBV and HDV infections.

Based on the above assumptions, we construct the model transmission diagram as shown in Fig 1.

The model construction is as follows.

**The susceptible individuals.** From assumption A2, all newborn are susceptible. Hence, we have all newborn will enter the population from the *S* compartment with a constant rate of Λ. Our model consider vaccination for HBV (see assumption A4), which is given at a constant rate *u*_1_. Hence, individuals from*S* who got vaccinated will goes to the *V* compartment. Since vaccination for HBV is not for life time, then after some periode, individuals form *V* will go back to *S* compartment when their vaccine effect vanished. We assume this drop out at a constant rate α. From assumption A3, HBV infection only occur from direct contact between susceptible and infected HBV individuals with a successful probability of infection is given by $\beta_{b}$. With *u*_2_ represent the liver transplantation effort, then the dynamic of the Susceptible compartment is given as follwos:


$$\begin{array}{c} \frac{dS}{dt}=\Lambda -(\beta_{b}I_{b}+u_{1}+\mu )S+u_{2}R+\alpha V. \end{array}$$

**The Vaccinated individuals.** From assumption A4, the number of vaccinated individuals increased only caused by vaccine intervention from *S* compartment with a constant rate *u*_1_. This group of individuals decreases due to natural death rate μ and caused by the effect of vaccine dissappeared with a rate of α. Since from assumption A4 the vaccine is not 100% effective to give protection from HBV, then vaccinated individuals may get infected by HBV caused by contact with $I_{b}$ with a probability of infection β reduced caused by vaccine efficacy, denoted by ξ. Hence, the dynamic of vaccinated compartment is given by:


$$\begin{array}{c} \frac{dV}{dt}=u_{1}S-((1-\xi )\beta_{b}I_{b}+\alpha +\mu )V. \end{array}$$

**The HBV infected individuals.** From the description of previous two compartment, newly HBV infection are coming from *S* and *V* compartment. Hence, the $I_{b}$ compartment will increase caused by new infection of $\beta_{b}SI_{b}+(1-\xi )\beta_{b}VI_{b}$. From assumption A5, infected individual $I_{b}$ only can get recovered from HBV if the follow the treatment. From assumption A7, treatment were assumed to be not always success to cure $I_{b}$ individuals. We assume the chances of successful treatment is *p* with a constant treatment rate γ. Hence, infected individual $I_{b}$ will go to recovered compartment *R* with a constant rate pγ, while the unsuccessfull proportion, with a rate of 1−p)γ, goes to chronic stage compartment *C*. Assuming the infected individual $I_{b}$ may death due to HBV with a constant rate $\delta_{b}$, then the dynamic of $I_{b}$ is given by:


$$\begin{array}{c} \frac{dI_{b}}{dt}=(S+(1-\xi )V)\beta_{b}I_{b}-(\gamma +\delta_{b}+\mu )I_{b}. \end{array}$$

**The Recovered individuals.** As previously mentioned in the construction of the dynamic of $I_{b}$, recovered individuals from $I_{b}$ will enter the recovered compartment *R* with a rate of pγ. This individuals still able to get infected by HDV if they do not conduct liver transplantation. Hence, if they conduct a liver transplantation with a constant rate *u*_2_, this individuals will come back to susceptible compartment. On the other hand, if they fo not conduct a liver transplantation, they will get infected by HDV if the contact with $I_{d}$ individuals with a constant infection rate $\beta_{d}$ (using assumption A8). Based on this description, the dynamic of recovered indivioduals is modeled using the following equation:


$$\begin{array}{c} \frac{dR}{dt}=p\gamma I_{b}-\beta_{d}RI_{d}-(u_{2}+\mu )R. \end{array}$$

**The Chronic individuals.** This group of individuals consist of infected HBV individuals, who already conduct a treatment for HBV, but failed to recover. Hence, this compartment increases due to treatment failure from $I_{b}$ compartment with a constant rate (1−p)γ. Furthermore, this compartment will decreases due to new infection with HDV virus with a constant rate $\beta_{d}$, natural death rate μ and death induced by HBV with a constant rate $\delta_{b}$. Hence, the dynamic of *C* compartment is given as follows:


$$\begin{array}{c} \frac{dC}{dt}=(1-p)\gamma I_{b}-(\beta_{d}C+\delta_{b}+\mu )C. \end{array}$$

**The HDV infected individuals.** This compartment increases due to new infection from *R* and *C* with a probability of infection of $\beta_{d}$, and decreases due to natural death rate μ and death induced by HDV with a constant rate $\delta_{d}$. We assume that HDV infected individual unable to recover due to it severe symptoms. Hence, the dynamic of $I_{d}$ compartment is given by:


$$\begin{array}{c} \frac{dI_{d}}{dt}=\beta_{d}(C+R)I_{d}-(\delta_{d}+\mu )I_{d}. \end{array}$$

Based on the above description, the mathematical model of Hepatitis B and Hepatitis D which consider the impact of vaccination and treatment failure integrated with liver transplantation is given by the following system of ordinary differential equation:


$$\begin{array}{cc} \frac{dS}{dt} & =\Lambda -(\beta_{b}I_{b}+u_{1}+\mu )S+u_{2}R+\alpha V, \end{array}$$ (1a)

$$\begin{array}{cc} \frac{dV}{dt} & =u_{1}S-((1-\xi )\beta_{b}I_{b}+\alpha +\mu )V, \end{array}$$ (1b)

$$\begin{array}{cc} \frac{dI_{b}}{dt} & =(S+(1-\xi )V)\beta_{b}I_{b}-(\gamma +\delta_{b}+\mu )I_{b}, \end{array}$$ (1c)

$$\begin{array}{cc} \frac{dR}{dt} & =p\gamma I_{b}-\beta_{d}RI_{d}-(u_{2}+\mu )R, \end{array}$$ (1d)

$$\begin{array}{cc} \frac{dC}{dt} & =(1-p)\gamma I_{b}-(\beta_{d}I_{d}+\delta_{b}+\mu )C, \end{array}$$ (1e)

$$\begin{array}{cc} \frac{dI_{d}}{dt} & =\beta_{d}(C+R)I_{d}-(\delta_{d}+\mu )I_{d}, \end{array}$$ (1f)

equipped with a non-negative initial condition:


$$\begin{array}{ccc} & & S(0)=S_{0}>0,V(0)=V_{0}\geq 0,I_{b}(0)=I_{b0}\geq 0,\\ & & R(0)=R_{0}\geq 0,C(0)=C_{0}\geq 0,I_{d}(0)=I_{d0}\geq 0. \end{array}$$

**Theorem 1.** *System (1) has a non-negative solutions if and only if the initial conditions are non-negative.*

**Proof 1** *We define the positive set as:*


$$\begin{array}{c} \Omega =\{(S,V,I_{b},R,C,I_{D})\in {\mathbb{R}}_{\geq 0}^{6}\}. \end{array}$$

*We will show that if the initial conditions of all variables lie in* Ω*, then the solution remains in* Ω *for all time* t≥0*. Observe that if a variable is zero, its rate of change remains non-negative:*


$$\begin{array}{cc} {\frac{dS}{dt}|}_{\begin{array}{c} S=0,\;V\geq 0,\;I_{b}\geq 0,\;R\geq 0,\;C\geq 0,\;I_{d}\geq 0 \end{array}} & =\Lambda +\alpha V+u_{2}R\geq 0, \end{array}$$ (2a)

$$\begin{array}{cc} {\frac{dV}{dt}|}_{\begin{array}{c} S\geq 0,\;V=0,\;I_{b}\geq 0,\;R\geq 0,\;C\geq 0,\;I_{d}\geq 0 \end{array}} & =u_{1}S\geq 0, \end{array}$$ (2b)

$$\begin{array}{cc} {\frac{dI_{b}}{dt}|}_{\begin{array}{c} S\geq 0,\;V\geq 0,\;I_{b}=0,\;R\geq 0,\;C\geq 0,\;I_{d}\geq 0 \end{array}} & =0, \end{array}$$ (2c)

$$\begin{array}{cc} {\frac{dR}{dt}|}_{\begin{array}{c} S\geq 0,\;V\geq 0,\;I_{b}\geq 0,\;R=0,\;C\geq 0,\;I_{d}\geq 0 \end{array}} & =p\gamma I_{b}\geq 0, \end{array}$$ (2d)

$$\begin{array}{cc} {\frac{dC}{dt}|}_{\begin{array}{c} S\geq 0,\;V\geq 0,\;I_{b}\geq 0,\;R\geq 0,\;C=0,\;I_{d}\geq 0 \end{array}} & =(1-p)\gamma I_{b}\geq 0, \end{array}$$ (2e)

$$\begin{array}{cc} {\frac{dI_{d}}{dt}|}_{\begin{array}{c} S\geq 0,\;V\geq 0,\;I_{b}\geq 0,\;R\geq 0,\;C\geq 0,\;I_{d}=0 \end{array}} & =0. \end{array}$$ (2f)

*From (2), all expressions are non-negative. Therefore, if the initial conditions are non-negative, any trajectory that tends toward the boundary will be repelled back into* Ω*. Hence, as long as the initial conditions are non-negative, the solution remains non-negative for all time.*

For the boundedness of the solution, we define the total population as:


$$\begin{array}{c} N(t)=S(t)+V(t)+I_{b}(t)+R(t)+C(t)+I_{b}(t). \end{array}$$

Summing all equations in system 1, we obtain:


$$\begin{array}{cc} \frac{dN}{dt} & =\frac{dS}{dt}+\frac{dV}{dt}+\frac{dI_{b}}{dt}+\frac{dR}{dt}+\frac{dC}{dt}+\frac{dI_{d}}{dt}\\ & =\Lambda -\mu (S+V+I_{b}+R+C+I_{d})-\delta_{b}(I_{b}+C)-\delta_{d}I_{d}\\ & =\Lambda -\mu N(t)-\delta_{d}(I_{b}+C)-\delta_{d}I_{d}\\ & \leq \Lambda -\mu N(t). \end{array}$$

By considering the differential inequality:


$$\begin{array}{c} \frac{dN}{dt}\leq \Lambda -\mu N(t), \end{array}$$

and comparing it with the auxiliary system:


$$\begin{array}{c} \frac{dy}{dt}=\Lambda -\mu y,\;y(0)=N(0), \end{array}$$

which has the explicit solution:


$$\begin{array}{c} y(t)=\frac{\Lambda }{\mu }+\left(N(0)-\frac{\Lambda }{\mu }\right)e^{-\mu t}. \end{array}$$

Thus, it follows that:


$$\begin{array}{c} N(t)\leq y(t)\leq \max\left\{N(0),\frac{\Lambda }{\mu }\right\},\;\forall t\geq 0. \end{array}$$

This implies that the total population *N*(*t*) is bounded. Since all compartmental variables are components of *N*(*t*) and are non-negative, it follows that:


$$\begin{array}{c} S(t),\;V(t),\;I_{b}(t),\;R(t),\;C(t),\;I_{d}(t) \end{array}$$

are also bounded. Therefore, the solution of system 1 is always non-negative and bounded for all t≥0.

In the next section, we analyze the model by investigating the existence and stability of its equilibrium points and deriving the basic reproduction number.

## 3. Model analysis

### 3.1. Disease free equilibrium and the basic reproduction number $\left({ℛ}_{0}\right)$

The disease-free equilibrium in an epidemiological model represents a state in which the population does not contain infected individuals. This equilibrium is obtained by setting the left-hand side of model 1 equal to zero, and solve it respect to all variables, by assuming the infected compartments are 0. This result stated in the following theorem.

**Theorem 2.** *The disease-free equilibrium of model 1 is given by:*


$$\begin{array}{c} \text{DFE}=(S^{0},V^{0},I_{b}^{0},R^{0},C^{0},I_{D}^{0})=\left(\frac{\Lambda (\alpha +\mu )}{\mu (\alpha +\mu +u_{1})},\frac{\Lambda u_{1}}{\mu (\alpha +\mu +u_{1})},0,0,0,0\right). \end{array}$$

*and it always exists.*

From the expression of *V*^0^, we can see that


$$\begin{array}{c} \frac{\partial V^{0}}{\partial u_{1}}=\frac{\Lambda (\alpha +\mu )}{\mu (\alpha +\mu +u_{1}{)}^{2}}>0, \end{array}$$

which indicates that the total number of vaccinated individuals at the disease-free equilibrium increases as the vaccination rate increases. On the other hand, since $S^{0}+V^{0}=\frac{\Lambda }{\mu }$, increasing the vaccination rate will reduce the size of the susceptible compartment. Furthermore, we have that $\frac{S^{0}}{V^{0}}=\frac{\alpha +\mu }{u_{1}}$. Hence, in an ideal situation, we would like this ratio to approach zero. This can be achieved either by increasing the vaccination rate *u*_1_ or by improving the duration of vaccine protection, which is equivalent to reducing α. This relationship also shows that relying solely on increasing vaccination coverage may not be sufficient if vaccine-induced immunity wanes rapidly, highlighting a trade-off between coverage and durability in determining the long-term effectiveness of vaccination strategies.

Before we analyze the stability of *DFE*, we will calculate the basic reproduction number of our model. The basic reproduction number (${ℛ}_{0}$) represents the expected number of secondary infections produced by a single infectious individual in a wholly susceptible population during in one infection period. We will use the Next-Generation Matrix method to calculate ${ℛ}_{0}$ [56].

First, we consider the infected compartments


$$\begin{array}{c} x=(I_{b},C,I_{d}{)}^{T}. \end{array}$$

Then the infected subsystem can be written as


$$\begin{array}{c} \frac{dx}{dt}=ℱ(x)-𝒱(x), \end{array}$$

where


$$\begin{array}{c} ℱ(x)=(\begin{array}{c} \beta_{b}(S+(1-\xi )V)I_{b}\\ 0\\ \beta_{d}(C+R)I_{d} \end{array}),\;𝒱(x)=(\begin{array}{c} (\gamma +\delta_{b}+\mu )I_{b}\\ (\beta_{d}I_{d}+\delta_{b}+\mu )C-(1-p)\gamma I_{b}\\ (\delta_{d}+\mu )I_{d} \end{array}). \end{array}$$

Evaluating the Jacobian matrices at the disease-free equilibrium


$$\begin{array}{c} E_{0}=(S^{0},V^{0},0,0,0,0), \end{array}$$

we obtain


$$\begin{array}{c} F=[\begin{array}{ccc} \beta_{b}(S^{0}+(1-\xi )V^{0}) & 0 & 0\\ 0 & 0 & 0\\ 0 & 0 & 0 \end{array}],\;V=[\begin{array}{ccc} \gamma +\delta_{b}+\mu & 0 & 0\\ -(1-p)\gamma & \delta_{b}+\mu & 0\\ 0 & 0 & \delta_{d}+\mu \end{array}]. \end{array}$$

Therefore, the basic reproduction number is


$$\begin{array}{c} {ℛ}_{0}=\rho (FV^{-1})=\frac{\beta_{b}(S^{0}+(1-\xi )V^{0})}{\gamma +\delta_{b}+\mu }. \end{array}$$

Substituting


$$\begin{array}{c} S^{0}=\frac{\Lambda (\alpha +\mu )}{\mu (\alpha +\mu +u_{1})},\;V^{0}=\frac{\Lambda u_{1}}{\mu (\alpha +\mu +u_{1})}, \end{array}$$

yields


$$\begin{array}{c} {ℛ}_{0}=\frac{\Lambda \beta_{b}(u_{1}-\xi u_{1}+\alpha +\mu )}{\mu (\alpha +\mu +u_{1})(\gamma +\delta_{b}+\mu )}. \end{array}$$ (3)

**Theorem 3.** *Let* ${ℛ}_{0}$ *be the basic reproduction number of system 1. Then the disease-free equilibrium (DFE) is locally asymptotically stable if* ${ℛ}_{0}<1$*, and unstable if* ${ℛ}_{0}>1$*.*

**Proof 2** *The Jacobian matrix of system (1) evaluated at DFE is given by*


$$\begin{array}{c} J_{DFE}=[\begin{array}{cc} J_{11} & J_{12}\\ J_{21} & J_{22} \end{array}], \end{array}$$

*where*


$$\begin{array}{c} J_{11}=[\begin{array}{ccc} -(\mu +u_{1}) & \alpha & a_{13}\\ u_{1} & -(\alpha +\mu ) & a_{23}\\ 0 & 0 & a_{33} \end{array}],\;J_{12}=[\begin{array}{ccc} u_{2} & 0 & 0\\ 0 & 0 & 0\\ 0 & 0 & 0 \end{array}], \end{array}$$

$$\begin{array}{c} J_{21}=[\begin{array}{ccc} 0 & 0 & p\gamma \\ 0 & 0 & (1-p)\gamma \\ 0 & 0 & 0 \end{array}],\;J_{22}=[\begin{array}{ccc} -(\mu +u_{2}) & 0 & 0\\ 0 & -(\mu +\delta_{b}) & 0\\ 0 & 0 & -(\mu +\delta_{d}) \end{array}], \end{array}$$

*with*


$$\begin{array}{c} \begin{array}{cc} a_{13} & =-\frac{\Lambda (\alpha +\mu )\beta_{b}}{\mu (\alpha +\mu +u_{1})},\\ a_{23} & =-\frac{(1-\xi )\beta_{b}\Lambda u_{1}}{\mu (\alpha +\mu +u_{1})},\\ a_{33} & =\frac{\Lambda (\alpha +\mu )\beta_{b}}{\mu (\alpha +\mu +u_{1})}+\frac{(1-\xi )\beta_{b}\Lambda u_{1}}{\mu (\alpha +\mu +u_{1})}-(\gamma +\delta_{b}+\mu ). \end{array} \end{array}$$

*The eigenvalues of* $J_{DFE}$ *are given by*


$$\begin{array}{c} \begin{array}{cc} \lambda_{1} & =(\gamma +\mu +\delta_{b})({ℛ}_{0}-1),\\ \lambda_{2} & =-(\mu +u_{2}),\\ \lambda_{3} & =-(\mu +\delta_{b}),\\ \lambda_{4} & =-(\mu +\delta_{d}),\\ \lambda_{5} & =-\mu ,\\ \lambda_{6} & =-(\alpha +\mu +u_{1}). \end{array} \end{array}$$

*Since all model parameters are positive, it follows that* $\lambda_{i}<0$ *for* i=2,...,6*. The sign of* $\lambda_{1}$ *depends on* ${ℛ}_{0}$*, and* $\lambda_{1}<0$ *whenever* ${ℛ}_{0}<1$*. Therefore, all eigenvalues have negative real parts if* ${ℛ}_{0}<1$*, and the DFE is locally asymptotically stable. Conversely, if* ${ℛ}_{0}>1$*, then* $\lambda_{1}>0$*, and the DFE is unstable.*

### 3.2. Existence of the endemic equilibrium points

An endemic equilibrium of the system is a steady-state solution of the form


$$\begin{array}{c} EE=(S^{+},V^{+},I_{b}^{+},R^{+},C^{+},I_{d}^{+}), \end{array}$$

where at least one infected compartment is positive. In this model, we consider two possible endemic equilibrium cases, namely:

- the HBV-only endemic equilibrium, where $I_{b}^{+}>0$ and $I_{d}^{+}=0$, which later called as *EE*_1_, and
- the HBV–HDV co-infection endemic equilibrium, where $I_{b}^{+}>0$ and $I_{d}^{+}>0$, which later called as *EE*_2_.

#### 3.2.1. Endemic equilibrium for $I_{d}=0$ (*EE*_1_).

We consider the endemic equilibrium corresponding to the persistence of HBV in the absence of HDV, that is, $I_{d}^{*}=0$. Under this assumption, the endemic equilibrium is given by:


$$\begin{array}{c} EE_{1}=(S,V,I_{b},R,C,I_{d})=(S^{*},V^{*},I_{b}^{*},R^{*},C^{*},I_{d}^{*}), \end{array}$$

where


$$\begin{array}{c} \begin{array}{cc} S^{*} & =\frac{\left(I_{b}^{*}(\xi -1)\beta_{b}-\mu -\alpha \right)(\gamma +\mu +\delta_{b})}{\left(I_{b}^{*}(\xi -1)\beta_{B}+(\xi -1)u_{1}-\mu -\alpha \right)\beta_{b}},\\ V^{*} & =\frac{u_{1}(\gamma +\mu +\delta_{b})}{\left(I_{b}^{*}(\xi -1)\beta_{b}+(\xi -1)u_{1}-\mu -\alpha \right)\beta_{b}},\\ R^{*} & =\frac{p\gamma I_{b}^{*}}{\mu +u_{2}},\\ C^{*} & =\frac{\mu^{2}+(u_{2}+\delta_{d})\mu -I_{b}^{*}\gamma p\beta_{d}+\delta_{d}u_{2}}{\beta_{d}(\mu +u_{2})},\\ I_{d}^{*} & =0, \end{array} \end{array}$$

where $I_{b}^{*}$ is obtained from the positive roots of the following quadratic equation:


$$\begin{array}{c} ℱ(I_{b})=A\beta^{2}I_{b}^{*2}+B\beta I_{b}^{*}+C({ℛ}_{0}-1)=0, \end{array}$$ (4)

with *A*, *B*, and *C* are constants depending on the model parameters and are given by:


$$\begin{array}{cc} A & =-((p-1)\gamma +\mu +\delta_{b})u_{2}-\mu (\gamma +\mu +\delta_{b})(\xi -1),\\ B & =(\xi -2)\mu^{3}+((\xi -2)u_{2}+(\delta_{b}+\gamma +u_{1})\xi -2\delta_{b}-\alpha -2\gamma -u_{1})\mu^{2}\\ & \;+(((\delta_{b}+\gamma +u_{1})\xi +(p-2)\gamma -2\delta_{b}-\alpha -u_{1})u_{2}+(\delta_{b}+\gamma )u_{1})\mu \\ & \;+\delta_{b}\alpha -\alpha \gamma +\Lambda \beta_{b}\mu \\ & \;-(((p-1)\gamma -\delta_{b})u_{1}+\Lambda \beta_{b})\xi +(1-p)\gamma +\delta_{b})u_{1}\\ & \;-(p-1)\alpha \gamma +\delta_{b}\alpha -\Lambda \beta_{b}u_{2},\\ C & =(\mu +u_{2})(\gamma +\mu +\delta_{b})(\alpha +\mu +u_{1})\mu . \end{array}$$ (5)

From the expression of *A*, it follows that *A* > 0 if


$$\begin{array}{c} p>1+\frac{\mu (\gamma +\mu +\delta_{b}+u_{2})+u_{2}\delta_{b}}{\gamma u_{2}}. \end{array}$$

However, since p∈[0,1], the above condition cannot be satisfied. Therefore, *A* < 0 for all biologically feasible parameter values.

Consequently, by Descartes’ Rule of Signs, the existence of a positive solution $I_{b}^{*}$ (and hence an endemic equilibrium for $I_{d}=0$) can be determined based on the signs of the coefficients, as summarized in Table 3.

**Table 3:** Descartes’ rule of signs for $I_{b}^{*}$.

Case	*A*	*B*	*C*	${ℛ}_{0}$	Number of positive roots
1	–	+	–	${ℛ}_{0}<1$	0 or 2
2	–	+	+	${ℛ}_{0}>1$	1
3	–	–	–	${ℛ}_{0}<1$	0
4	–	–	+	${ℛ}_{0}>1$	1

Table 3 shows that the existence and number of endemic equilibria depend on the value of the basic reproduction number ${ℛ}_{0}$. When ${ℛ}_{0}<1$, the system admits either zero or two positive roots, corresponding to the absence or multiplicity of endemic equilibria. In contrast, when ${ℛ}_{0}>1$, the system admits exactly one positive root, implying the existence of a unique endemic equilibrium. This result stated in the following lemma.

**Lemma 1.** *The HBV-HDV model in system (1) admits a unique endemic equilibrium EE*_*1*_ *when* ${ℛ}_{0}>1$*.*

Theorem 1 guarantee the existence of *EE*_1_ whenever ${ℛ}_{0}>1$. However, from Table 3, system (1) may have 2 *EE*_1_ when ${ℛ}_{0}<1$. Hence, it is important to analyze the direction of polynomial $ℱ(I_{b})$ at ${ℛ}_{0}=1,I_{b}=0$. If the gradient is positive, then we will have no endemic equilibrium when ${ℛ}_{0}<1$, and vice versa if the gradient is negative.

#### 3.2.2. Gradient analysis for $I_{d}=0$.

From equation 3, the transmission parameter can be expressed as


$$\begin{array}{c} \beta_{b}^{*}=\frac{\mu (\alpha +\mu +u_{1})(\gamma +\delta_{b}+\mu )}{\Lambda (u_{1}-\xi u_{1}+\alpha +\mu )}. \end{array}$$ (6)

Substituting 6 into equation (4) and (5), we obtain the following quadratic equation:


$$\begin{array}{c} F(I_{b}^{*},{ℛ}_{0})=a({ℛ}_{0})(I_{b}^{*}{)}^{2}+b({ℛ}_{0})I_{b}^{*}+c({ℛ}_{0})=0. \end{array}$$

where


$$\begin{array}{c} \begin{array}{cc} a({ℛ}_{0}) & =\frac{(\xi -1)G^{2}E^{2}\mu^{2}Q}{\Lambda^{2}D^{2}}{ℛ}_{0}^{2},\\ b({ℛ}_{0}) & =\frac{{ℛ}_{0}}{\Lambda D}\left[\frac{\mu EG}{D}\left(((1-\xi )\mu -\xi )u_{2}+1\right)+P-\mu EG\right],\\ c({ℛ}_{0}) & =\mu (\mu +u_{2})GE(1-{ℛ}_{0}), \end{array} \end{array}$$

with


$$\begin{array}{c} \begin{array}{cc} D & :=\alpha +\mu +(1-\xi )u_{1},\\ E & :=\alpha +\mu +u_{1},\\ G & :=\gamma +\delta_{b}+\mu ,\\ H & :=(1-p)\gamma +\delta_{b},\\ Q & :=\mu^{2}+(\gamma +\delta_{b}+u_{2})\mu +Hu_{2}. \end{array} \end{array}$$

and


$$\begin{array}{c} \begin{array}{cc} P:= & (\xi -2)\mu^{3}\\ & +[(\xi -2)u_{2}+\xi (\delta_{b}+\gamma +u_{1})-2\delta_{b}-\alpha -2\gamma -u_{1}]\mu^{2}\\ & +[(\xi (\delta_{b}+\gamma +u_{1})-u_{1}+(p-2)\gamma -2\delta_{b}-\alpha )u_{2}\\ & \;+(\xi -1)(\delta_{b}+\gamma )u_{1}-\alpha (\delta_{b}+\gamma )]\mu \\ & -[(p-1)\gamma -\delta_{b}]u_{1}\xi -Hu_{1}+\alpha [(p-1)\gamma +\delta_{b}]u_{2}. \end{array} \end{array}$$

Next, differentiating with respect to ${ℛ}_{0}$, we obtain


$$\begin{array}{c} \frac{\partial a({ℛ}_{0})}{\partial {ℛ}_{0}}(I_{b}^{*}{)}^{2}+2I_{b}^{*}\frac{\partial I_{b}^{*}}{\partial {ℛ}_{0}}a({ℛ}_{0})+\frac{\partial b({ℛ}_{0})}{\partial {ℛ}_{0}}I_{b}^{*}+\frac{\partial I_{b}^{*}}{\partial {ℛ}_{0}}b({ℛ}_{0})+\frac{\partial c({ℛ}_{0})}{\partial {ℛ}_{0}}=0. \end{array}$$

Substituting $I_{b}^{*}=0$, we obtain:


$$\begin{array}{c} \begin{array}{cc} \frac{\partial (I_{b}^{*})}{\partial {ℛ}_{0}}\;b({ℛ}_{0})+\frac{\partial c({ℛ}_{0})}{\partial {ℛ}_{0}} & =0,\\ -\frac{\partial (I_{b}^{*})}{\partial {ℛ}_{0}}\;b({ℛ}_{0}) & =\frac{\partial c({ℛ}_{0})}{\partial {ℛ}_{0}},\\ \frac{\partial (I_{b}^{*})}{\partial {ℛ}_{0}} & =-\frac{\partial c({ℛ}_{0})}{\partial {ℛ}_{0}}\cdot \frac{1}{b({ℛ}_{0})}. \end{array} \end{array}$$

Based on the explanation above, the partial derivative of $I_{b}^{*}$ with respect to ${ℛ}_{0}$ is given by:


$$\begin{array}{cc} \frac{\partial (I_{b}^{*})}{\partial {ℛ}_{0}} & =\mu^{4}+\left[(\xi^{2}-3\xi +2)u_{1}+2\alpha +\gamma +u_{2}+\delta_{b}\right]\mu^{3}\\ & \;+[(-1+\xi {)}^{2}u_{1}^{2}+((\xi -2)u_{2}+(\delta_{b}+\gamma )\xi -2\alpha -2\gamma -2\delta_{b})(-1+\xi )u_{1}\\ & \;+((1-p)\gamma +2\alpha +\delta_{b})u_{2}+\alpha (\alpha +2\gamma +2\delta_{b})]\mu^{2}\\ & \;+[(-1+\xi {)}^{2}(u_{2}+\delta_{b}+\gamma )u_{1}^{2}\\ & \;+2(\left(\frac{(\delta_{b}+\gamma )\xi }{2}+\gamma (p-1)-\alpha -\delta_{b}\right)u_{2}-\alpha (\delta_{b}+\gamma ))(-1+\xi )u_{1}\\ & \;-2\alpha \left((\gamma (p-1)-\frac{\alpha }{2}-\delta_{b})u_{2}-\frac{\alpha (\delta_{b}+\gamma )}{2}\right)]\mu \\ & \;-(\gamma (p-1)-\delta_{b})u_{2}(-\alpha +(-1+\xi )u_{1}{)}^{2}. \end{array}$$ (7)

Equation (7) is positive if $p>p^{*}$, where:


$$\begin{array}{c} p^{*}=\frac{(\mu +u_{2})\left(\mu^{2}+((\xi^{2}-3\xi +2)u_{1}+2\alpha )\mu +(-\alpha +(-1+\xi )u_{1}{)}^{2}(\gamma +\delta_{b}+\mu )\right)}{\gamma u_{2}{\left(\mu +(1-\xi )u_{1}+\alpha \right)}^{2}}. \end{array}$$ (8)

However, the expression of $p^{*}$ always tends to be larger than one, which is contrary to the condition of p∈[0,1]. Therefore, eventhough a condition of $\frac{\partial I_{b}}{\partial {ℛ}_{0}}<0$ is possible mathematically, but biologically this is not the case. Hence, we ignore the case of the existence of another *EE*_1_ when ${ℛ}_{0}<1$. Based on this results, we have the following theorem.

**Theorem 4.** *The HBV-HDV model in system (1) only have a unique endemic equilibrium EE*_*1*_ *if* ${ℛ}_{0}>1$*, and no endemic equilibrium with* $I_{d}=0$ *otherwise.*

#### 3.2.3. Stability analysis of the endemic equilibrium point when $I_{d}=0$.

In this subsection, we analyze the stability of the endemic equilibrium point when $I_{d}=0$ using the Castillo-Chavez and Song theorem. The system of equations when $I_{d}=0$ can be written as follows:


$$\begin{array}{cc} \frac{dS}{dt} & =\Lambda +\alpha V+u_{2}R-(u_{1}+\beta_{b}(I_{b})+\mu )S, \end{array}$$ (9a)

$$\begin{array}{cc} \frac{dV}{dt} & =u_{1}S-\left(\alpha +\mu +(1-\xi )\beta_{b}(I_{b})\right)V, \end{array}$$ (9b)

$$\begin{array}{cc} \frac{dI_{b}}{dt} & =\left((1-\xi )V+S\right)\beta_{b}(I_{b})-(p\gamma +\delta_{b}+\mu )(I_{b}), \end{array}$$ (9c)

$$\begin{array}{cc} \frac{dR}{dt} & =p\gamma (I_{b})-(u_{2}+\mu )R, \end{array}$$ (9d)

$$\begin{array}{cc} \frac{dC}{dt} & =(1-p)\gamma (I_{b})-(\delta_{b}+\mu )C. \end{array}$$ (9e)

Next, choose the parameter $\beta_{b}$ for the case ${ℛ}_{0}=1$ so that the equation can be rewritten as follows:


$$\begin{array}{c} \beta_{b}^{*}=\frac{\mu \left(\alpha \gamma +\alpha \mu +\alpha \delta_{b}+\gamma \mu +\gamma u_{1}+\mu^{2}+\mu \delta_{b}+\mu u_{1}+\delta_{b}u_{1}\right)}{\Lambda (-\xi u_{1}+\alpha +\mu +u_{1})}. \end{array}$$

The Jacobian matrix of system (9) is:


$$\begin{array}{c} J=[\begin{array}{ccccc} \mu -u_{1} & \alpha & a_{13} & u_{2} & 0\\ u_{1} & -\alpha -\mu & a_{23} & 0 & 0\\ 0 & 0 & a_{33} & 0 & 0\\ 0 & 0 & p\gamma & -\mu -u_{2} & 0\\ 0 & 0 & (1-p)\gamma & 0 & -\delta_{b}-\mu \end{array}], \end{array}$$

where


$$\begin{array}{c} \begin{array}{cc} a_{13} & =-\frac{\left(\alpha \gamma +\alpha \mu +\alpha \delta_{b}+\gamma \mu +\gamma u_{1}+\mu^{2}+\mu \delta_{b}+\mu u_{1}+\delta_{b}u_{1})(\alpha +\mu \right)}{\left(-\xi u_{1}+\alpha +\mu +u_{1})(\alpha +\mu +u_{1}\right)},\\ a_{23} & =-\frac{(1-\xi )(\alpha \gamma +\alpha \mu +\alpha \delta_{b}+\gamma \mu +\gamma u_{1}+\mu^{2}+\mu \delta_{b}+\mu u_{1}+\delta_{b}u_{1})u_{1}}{\left(-\xi u_{1}+\alpha +\mu +u_{1})(\alpha +\mu +u_{1}\right)},\\ a_{33} & =\frac{\left(\alpha \gamma +\alpha \mu +\alpha \delta_{b}+\gamma \mu +\gamma u_{1}+\mu^{2}+\mu \delta_{b}+\mu u_{1}+\delta_{b}u_{1})(\alpha +\mu \right)}{\left(-\xi u_{1}+\alpha +\mu +u_{1})(\alpha +\mu +u_{1}\right)}\\ & \;+\frac{(1-\xi )(\alpha \gamma +\alpha \mu +\alpha \delta_{b}+\gamma \mu +\gamma u_{1}+\mu^{2}+\mu \delta_{b}+\mu u_{1}+\delta_{b}u_{1})u_{1}}{\left(-\xi u_{1}+\alpha +\mu +u_{1})(\alpha +\mu +u_{1}\right)}\\ & \;-p\gamma -(1-p)\gamma -\delta_{b}-\mu . \end{array} \end{array}$$

Hence, the eigenvalues are given by:


$$\begin{array}{cc} \lambda_{1} & =0,\\ \lambda_{2} & =-\mu -u_{2},\\ \lambda_{3} & =-\delta_{b}-\mu ,\\ \lambda_{4} & =-\mu ,\\ \lambda_{5} & =-\alpha -\mu -u_{1}. \end{array}$$

Next, by solving for the right eigenvector, we obtain:


$$\begin{array}{c} w=[v_{1}\;v_{2}\;v_{3}\;v_{4}\;v_{5}{]}^{T} \end{array}$$

with


$$\begin{array}{cc} w_{1} & =-\mu^{4}+(-2\alpha -\gamma -u_{2}-\delta_{b})\mu^{3}\\ & \;+\left(-\alpha^{2}+(-2u_{2}+(-1+\xi )u_{1}-2\gamma -2\delta_{b})\alpha +u_{2}((-1+p)\gamma -\delta_{b})\right)\mu^{2}\\ & \;+((-\gamma -u_{2}-\delta_{b})\alpha^{2}+(((-1+\xi )u_{1}+(2p-2)\gamma -2\delta_{b})u_{2}+u_{1}(\gamma +\delta_{b})(-1+\xi ))\alpha \\ & \;-p\gamma u_{1}u_{2}(-1+\xi ))\mu -(-\alpha +(-1+\xi )u_{1})\alpha u_{2}((-1+p)\gamma -\delta_{b}),\\ w_{2} & =u_{1}[(\xi -2)\mu^{3}+((-1+\xi )u_{1}+(\xi -2)u_{2}+(\gamma +\delta_{b})\xi -\alpha -2\gamma -2\delta_{b})\mu^{2}\\ & \;+((\gamma +u_{2}+\delta_{b})(-1+\xi )u_{1}+((\gamma +\delta_{b})\xi +(p-2)\gamma -\alpha -2\delta_{b})u_{2}-\alpha (\gamma +\delta_{b}))\mu \\ & \;-(-\alpha +(-1+\xi )u_{1})u_{2}((-1+p)\gamma -\delta_{b})],\\ w_{3} & =(\mu +u_{2})\;\mu (\alpha +\mu +u_{1})(\mu +\alpha +(1-\xi )u_{1}),\\ w_{4} & =\mu (\alpha +\mu +u_{1})(\mu +\alpha +(1-\xi )u_{1})p\gamma ,\\ w_{5} & =-\frac{(-1+p)(\mu +u_{2})\;\mu (\alpha +\mu +u_{1})(\mu +\alpha +(1-\xi )u_{1})p\gamma }{(\delta_{b}+\mu )p}. \end{array}$$

And the left eigenvector is obtained as


$$\begin{array}{c} v=[v_{1}\;v_{2}\;v_{3}\;v_{4}\;v_{5}]=[0\;0\;1\;0\;0]. \end{array}$$

#### 3.2.4. Determination of the value of *a.*

Since all components of the left eigenvector are zero except for *v*_3_, the derivative is computed only for terms involving the variable *v*_3_. Thus, the value of *a* is obtained as follows:


$$\begin{array}{cc} a & =v_{3}w_{3}w_{1}\frac{\partial^{2}(I_{b}^{*})}{\partial S\;\partial t}+v_{3}w_{3}w_{2}\frac{\partial^{2}(I_{b}^{*})}{\partial V\;\partial t}\\ & =[-\mu^{4}+\left((-\xi^{2}+3\xi -2)u_{1}-2\alpha -\gamma -u_{2}-\delta_{b}\right)\mu^{3}\\ & \;+(-(\xi -1{)}^{2}u_{1}^{2}-((\xi -2)u_{2}+(\gamma +\delta_{b})\xi -2\delta_{b}-2\alpha -2\gamma )(\xi -1)u_{1}\\ & \;+((-1+p)\gamma -2\alpha -\delta_{b})u_{2}-2(\delta_{b}+\frac{\alpha }{2}+\gamma )\alpha )\mu^{2}\\ & \;+(-(\xi -1{)}^{2}(\gamma +u_{2}+\delta_{b})u_{1}^{2}\\ & \;-2(\xi -1)(\left(\frac{(\gamma +\delta_{b})\xi }{2}+(-1+p)\gamma -\delta_{b}-\alpha \right)u_{2}-\alpha (\gamma +\delta_{b}))u_{1}\\ & \;+2\alpha (((-1+p)\gamma -\delta_{b}-\frac{\alpha }{2})u_{2}-\frac{\alpha (\gamma +\delta_{b})}{2}))\mu \\ & \;+((-1+p)\gamma -\delta_{b})(-\alpha +(\xi -1)u_{1}{)}^{2}u_{2}]\\ & \;\cdot (\mu +u_{2})\;\beta_{b}\;\mu (\alpha +\mu +u_{1})(\mu +\alpha +(1-\xi )u_{1}). \end{array}$$

The value of *a* will be positive if


$$\begin{array}{c} p^{*}=\frac{(\mu +u_{2})\left(\mu^{2}+((\xi^{2}-3\xi +2)u_{1}+2\alpha )\mu +(-\alpha +(-1+\xi )u_{1}{)}^{2}(\gamma +\delta_{b}+\mu )\right)}{\gamma u_{2}{\left(\mu +(1-\xi )u_{1}+\alpha \right)}^{2}}. \end{array}$$

This condition is the same as the condition for the existence of the endemic equilibrium when ${ℛ}_{0}<1$ in equation (8). Hence, we will always have *a* < 0.

#### 3.2.5. Determination of the value of *b.*

To determine the value of *b*, similar to *a*, only derivatives involving the variable *v*_3_ are considered. Thus, the value of *b* is obtained as follows:


$$\begin{array}{c} b=v_{3}w_{3}\frac{\partial^{2}(I_{b}^{*})}{\partial (I_{b}^{*}{)}^{2}}=\Lambda (-\xi u_{1}+\alpha +\mu +u_{1})(\mu +u_{2})(\mu +\alpha +(1-\xi )u_{1}). \end{array}$$ (10)

From equation (10), it can be seen that *b* is always positive. Therefore, the stability of $I_{d}=0$ can be concluded as follows:

**Theorem 5.** *When* $I_{d}=0$*, a forward bifurcation occurs always occurs at* ${ℛ}_{0}=1$*.*

#### 3.2.6. Endemic equilibrium when $I_{d}\neq 0$ (*EE*_2_).

The endemic equilibrium point in this case is an equilibrium point where there are individuals infected with HDV ($I_{d}\neq 0$), given by:


$$\begin{array}{c} EE_{2}=(S,V,I_{b},R,C,I_{d})=(S^{†},V^{†},I_{b}^{†},R^{†},C^{†},I_{d}^{†}), \end{array}$$

with


$$\begin{array}{cc} S^{†} & =\frac{S_{1}^{†}}{S_{2}^{†}},\;V^{†}=\frac{V_{1}^{†}}{V_{2}^{†}},\\ I_{b}^{†} & =\frac{\left(\mu +\delta_{d})((I_{d}^{†})\beta_{d}+\mu +\delta_{b})((I_{d}^{†})\beta_{d}+\mu +u_{2}\right)}{\beta_{d}\gamma ((\delta_{b}-u_{2})p+(I_{d}^{†})\beta_{d}+\mu +u_{2})},\\ R^{†} & =\frac{p(\mu +\delta_{d})((I_{d}^{†})\beta_{d}+\mu +\delta_{b})}{\beta_{d}((\delta_{b}-u_{2})p+(I_{d}^{†})\beta_{d}+\mu +u_{2})},\\ C^{†} & =\frac{\left(\mu +\delta_{d})((I_{d}^{†})\beta_{d}+\mu +u_{2})(p-1\right)}{\beta_{d}((\delta_{b}-u_{2})p+(I_{d}^{†})\beta_{d}+\mu +u_{2})}. \end{array}$$

and


$$\begin{array}{cc} S_{1}^{†} & =(\gamma +\delta_{b}+\mu )((1-\xi )((I_{d}^{†})\beta_{d}+\mu +u_{2})(\mu +\delta_{d}))\\ & \;\times ((I_{d}^{†})\beta_{d}+\mu +\delta_{b})\beta_{b}+\gamma (\alpha +\mu )\\ & \;\times (\mu +(I_{d}^{†})\beta_{d}+(1-p)u_{2}+p\delta_{b})\beta_{d},\\ S_{2}^{†} & =(1-\xi )((I_{d}^{†})\beta_{d}+\mu +u_{2})(\mu +\delta_{d})\\ & \;\times ((I_{d}^{†})\beta_{d}+\mu +\delta_{b})\beta_{b}+(-\xi u_{1}+\alpha +\mu +u_{1})\\ & \;\times \gamma (\mu +(I_{d}^{†})\beta_{d}+(1-p)u_{2}+p\delta_{b})\beta_{d},\\ V_{1}^{†} & =\gamma (\mu +(I_{d}^{†})\beta_{d}+(1-p)u_{2}+p\delta_{b})\\ & \;\times (\gamma +\delta_{b}+\mu )u_{1}\beta_{d},\\ V_{2}^{†} & =-(((I_{d}^{†})(\xi -1)\beta_{b}-\gamma )\mu +\delta_{d}(I_{d}^{†})(\xi -1)\beta_{b}\\ & \;+\gamma (\xi u_{1}-\alpha -u_{1})(I_{d}^{†})\beta_{d}^{2}\\ & \;+(-2(I_{d}^{†})(\xi -1)\beta_{b}+\gamma )\mu^{2}\\ & \;+(I_{d}^{†})(\xi -1)(\delta_{b}+2\delta_{d}+u_{2})\beta_{b}\\ & \;+\gamma ((1-p)u_{2}+p\delta_{b}-u_{1}\xi +\alpha +u_{1})\mu \\ & \;-\delta_{d}(I_{d}^{†})(\xi -1)(\delta_{b}+u_{2})\beta_{b}\\ & \;-\gamma ((1-p)u_{2}+p\delta_{b})(\xi u_{1}-\alpha -u_{1})\beta_{d}\\ & \;-\beta_{b}(\xi -1)(\mu +u_{2})(\mu +\delta_{d})(\delta_{b}+\mu ))\beta_{b}. \end{array}$$

while $\left(I_{d}^{†}\right)$ is taken from the positive roots of the following polynomial


$$\begin{array}{c} a_{4}I_{d}^{4}+a_{3}I_{d}^{3}+a_{2}I_{d}^{2}+a_{1}I_{d}+a_{0}=0 \end{array}$$

where expression of $a_{i}$ for *i* = 0,1,2,3,4 can be seen in S1 File.

To determine the number of positive roots of the polynomial


$$\begin{array}{c} P(I_{d})=a_{4}I_{d}^{4}+a_{3}I_{d}^{3}+a_{2}I_{d}^{2}+a_{1}I_{d}+a_{0}=0, \end{array}$$

we apply Descartes’ Rule of Signs. Under the biologically feasible assumptions that all parameters are positive and 0<ξ<1, the leading coefficient *a*_4_ is strictly negative. The signs of *a*_3_, *a*_2_, *a*_1_, and *a*_0_ depend on combinations of the model parameters. Therefore, the number of positive real roots of $P(I_{d})$ is given by the number of sign changes in the sequence $\left(a_{4},a_{3},a_{2},a_{1},a_{0}\right)$, or less than it by an even integer.

From the possible sign patterns summarized in Table 4, the polynomial may admit zero, one, two, three, or four positive real roots. In particular, if there are no sign changes, then the polynomial has no positive real roots, and hence no endemic equilibrium with $I_{d}>0$ exists. When there is exactly one sign change, the polynomial admits a unique positive real root. This implies the existence of a single endemic equilibrium with $I_{d}>0$. When multiple sign changes occur, the polynomial may admit multiple positive real roots. Thus, indicating the possibility of multiple endemic equilibria, when of course the existence of *EE*_2_ will depend on the positive values of other variables in *EE*_2_, not only $I_{d}^{†}$, i.e., $S^{†},V^{†},I_{b}^{†},R^{†}$ and $C^{†}$.

**Table 4:** Possible sign patterns of the coefficients of $P(I_{d})$ and the corresponding number of positive real roots according to Descartes’ Rule of Signs.

Case	*a*_4_	*a*_3_	*a*_2_	*a*_1_	*a*_0_	Sign Changes	# Positive Roots
1	–	+	+	+	+	1	1
2	–	+	+	+	–	2	0 or 2
3	–	+	+	–	+	3	1 or 3
4	–	+	+	–	–	2	0 or 2
5	–	+	–	+	+	3	1 or 3
6	–	+	–	+	–	4	0, 2, or 4
7	–	+	–	–	+	3	1 or 3
8	–	+	–	–	–	2	0 or 2
9	–	–	+	+	+	1	1
10	–	–	+	+	–	2	0 or 2
11	–	–	+	–	+	3	1 or 3
12	–	–	+	–	–	2	0 or 2
13	–	–	–	+	+	1	1
14	–	–	–	+	–	2	0 or 2
15	–	–	–	–	+	1	1
16	–	–	–	–	–	0	0

The condition


$$\begin{array}{c} \beta_{d}(C^{†}+R^{†})=\delta_{d}+\mu \end{array}$$

is obtained from the equilibrium of the $I_{d}$ – equation under $I_{d}\neq 0$. Since the compartments *R* and *C* are generated from HBV-infected individuals, a necessary condition for $C^{†}+R^{†}>0$ is that HBV persists in the population, that is, ${ℛ}_{0}>1$. Thus, ${ℛ}_{0}>1$ is a prerequisite for the existence of HDV infection. Under this condition, substituting the expressions of $R^{†}$ and $C^{†}$ into the above relation yields an algebraic equation involving both $I_{d}$ and $\beta_{d}$, which can be rearranged into a quadratic form in $\beta_{d}$ and solved explicitly. The resulting threshold $\beta_{d}^{*}$ represents the critical HDV transmission rate above which the algebraic equation admits positive solutions for $I_{d}^{†}$. Consequently, $\beta_{d}^{*}$ defines the boundary between the absence and persistence of HDV infection, and the endemic equilibrium with $I_{d}^{†}>0$ exists if and only if both ${ℛ}_{0}>1$ and $\beta_{d}>\beta_{d}^{*}$ are satisfied, where the expression of $\beta_{d}^{*}$ is given in S2 File.

These results suggest that the model may exhibit complex dynamical behavior, including the coexistence of multiple endemic states depending on parameter values. However, since the coefficients $a_{i}$ are highly nonlinear functions of the model parameters, it is generally difficult to determine analytically the exact number of positive roots. Therefore, numerical continuation methods are employed to further investigate the existence and stability of endemic equilibria.

## 4. Numerical experiments

### 4.1. Continuation results with *Matcont*

In this subsection, we start our numerical experiment with the purpose of analyzing the bifurcation diagram of system (1). All bifurcation diagrams were produced using *MatCont*, which is a MATLAB-based numerical tool for continuation and bifurcation analysis of dynamical systems. It enables the tracking of equilibria and periodic solutions as parameters vary, and detects bifurcations such as saddle-node, Hopf, and limit points, providing valuable insight into system stability and qualitative behavior.

First, we calculate the possible branches of equilibrium using the parameter values given in Table 2 and the initial condition


$$\begin{array}{c} (S(0),V(0),I_{b}(0),R(0),C(0),I_{d}(0))=(9900,0,850,50,50,50). \end{array}$$

**Fig 2:**
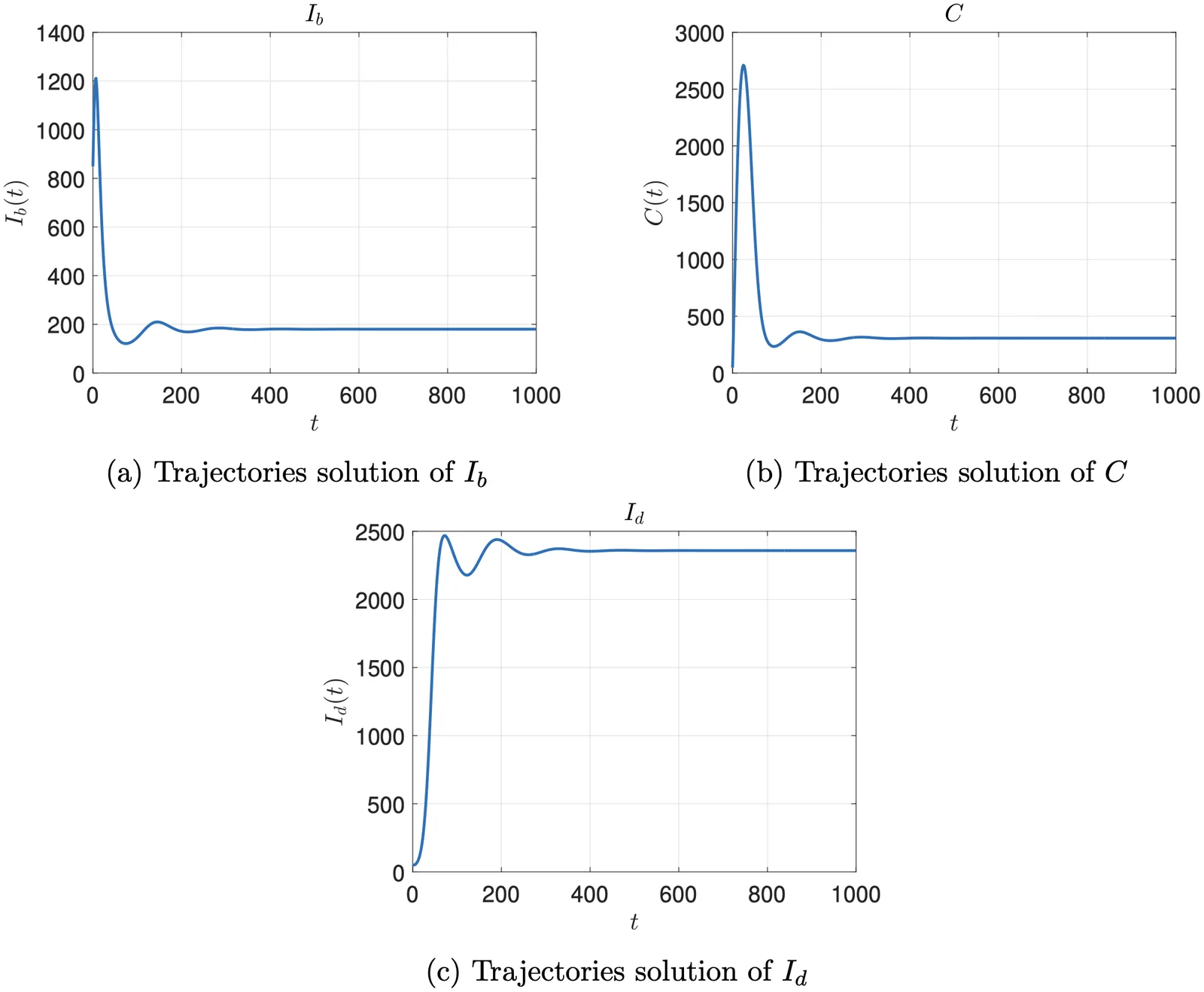
Trajectories of solution of system (1) with respect to $I_{b},C,$ and $I_{d}$. This figures show how variable $I_{b},C,$ and $I_{d}$ changes as times increases.

The system was solved numerically using an adaptive Runge–Kutta method of order 4(5) (RK45), selected for its established accuracy and efficiency for non-stiff systems of ordinary differential equations. The solutions are depicted in Fig 2, which shows the dynamics of $I_{b}$, *C*, and $I_{d}$, all of which tend to a stable endemic equilibrium. With these parameter values, we obtain ${ℛ}_{0}=1.4$, which yields three types of equilibrium.

The first equilibrium is the disease-free equilibrium (DFE),


$$\begin{array}{c} DFE=(S,V,I_{b},R,C,I_{d})=(9913,87,0,0,0,0), \end{array}$$

which is unstable. The second equilibrium is the endemic equilibrium when $I_{d}=0$, i.e.,


$$\begin{array}{c} EE_{1}=(S,V,I_{b},R,C,I_{d})=(7625,66,166,232,1651,0), \end{array}$$

which is also unstable. The third equilibrium is the endemic equilibrium when $I_{d}>0$, given by


$$\begin{array}{c} EE_{2}=(S,V,I_{b},R,C,I_{d})=(7625,66,136,131,325,1540), \end{array}$$

which is the only stable equilibrium for these parameter values.

**Fig 3:**
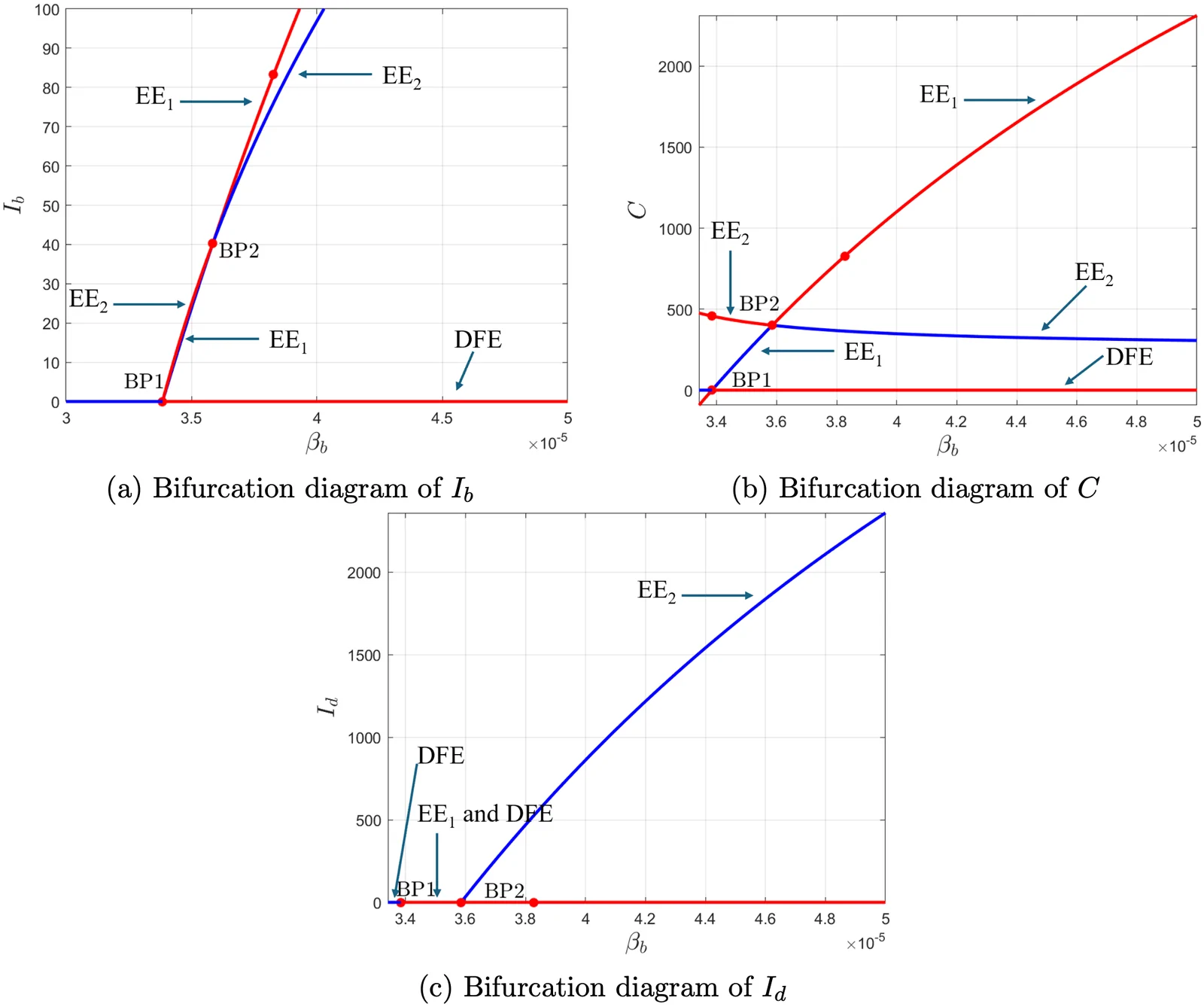
Bifurcation diagram of $I_{b},C,$ and $I_{d}$ with $\beta_{b}$ as the bifurcation parameter. The red and blue curve represent the unstable and stable equilibrium points. BP1 and BP2 represent the branching point.

Using these three equilibria, we track their branches of equilibria, and the results are depicted in Fig 3, using $\beta_{b}$ as the bifurcation parameter. From Fig 3, we identify two branching points, namely BP1 and BP2, corresponding to $\beta_{b}=3.38\times 10^{-5}$ and $\beta_{b}=3.58\times 10^{-5}$, respectively. When $\beta_{b}$ is smaller than BP1, we observe that the *DFE* is stable, while *EE*_1_ and *EE*_2_ do not exist. This stability structure is maintained as $\beta_{b}$ increases until it reaches BP1. When $\beta_{b}$ passes BP1, the *DFE* loses its stability, while *EE*_1_ and *EE*_2_ start to emerge. However, only *EE*_1_ is stable, while *EE*_2_ remains unstable. The stability of *EE*_1_ is maintained until $\beta_{b}$ reaches the second branching point, BP2. When $\beta_{b}$ passes BP2, *EE*_1_ loses its stability, while *EE*_2_ becomes stable. The stability of *EE*_2_ is then maintained for all $\beta_{b}$ larger than BP2.

**Fig 4:**
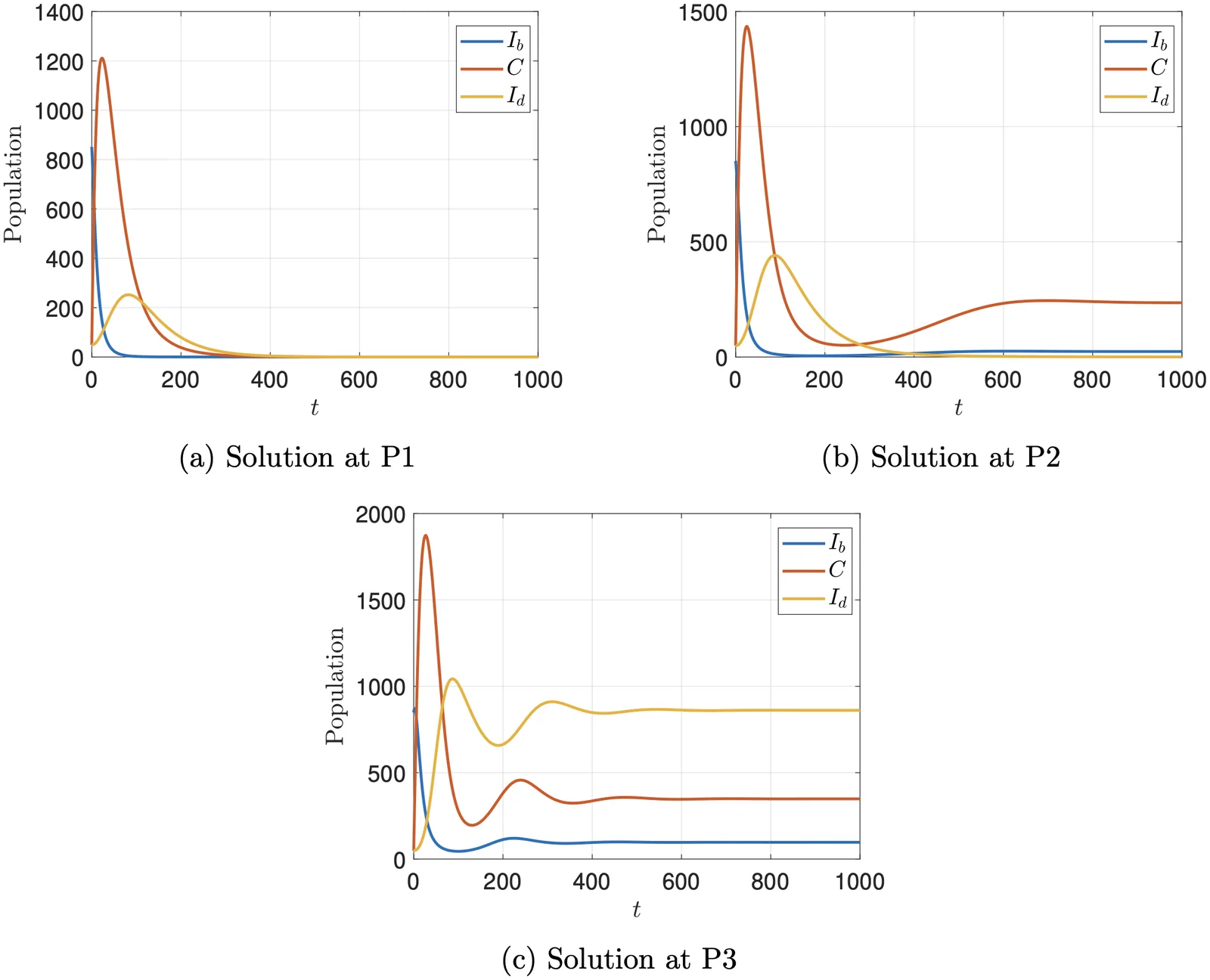
Three solution of system (1) with different values of $\beta_{h}$. Trajectories of solution of $I_{b},C,$ and $I_{d}$ with variation value of infection rate.

To illustrate this stability diagram in the solution of system (1) as shown in Fig 3, we choose three different sample points of $\beta_{b}$, namely point P1 at $\beta_{b}=3.2\times 10^{-5}$ ($\beta_{b}<\text{BP1}$), point P2 at $\beta_{b}=3.5\times 10^{-5}$ ($\beta_{b}\in [\text{BP1},\text{BP2}]$), and point P3 at $\beta_{b}=4\times 10^{-5}$ ($\beta_{b}>\text{BP2}$). The results are depicted in Fig 4. From Fig 4(a), we can clearly see that the system tends to the *DFE* when $\beta_{b}$ is chosen at P1. The system tends to *EE*_1_ when $\beta_{b}$ is chosen at P2, as shown in Fig 4(b), and tends to *EE*_2_ when $\beta_{b}$ is chosen at P3, as shown in Fig 4(c).

### 4.2. Global sensitivity analysis

The next numerical experiment here is the global sensitivity analysis of ${ℛ}_{0}$ and the variables in system (1) with Partial Rank Corellation Coefficient (PRCC) combined with Latin Hypercube Sampling. This method was introduced by authors in [57], and after that widely used in many epidemic models, including our previous works in [26,58,59]. The PRCC analysis conducted using baseline parameters in Table 2 with sampling range taking from ±25% of the baseline values, except *u*_1_ which taken between 0 and 0.1, with 50000 sample points.

**Fig 5:**
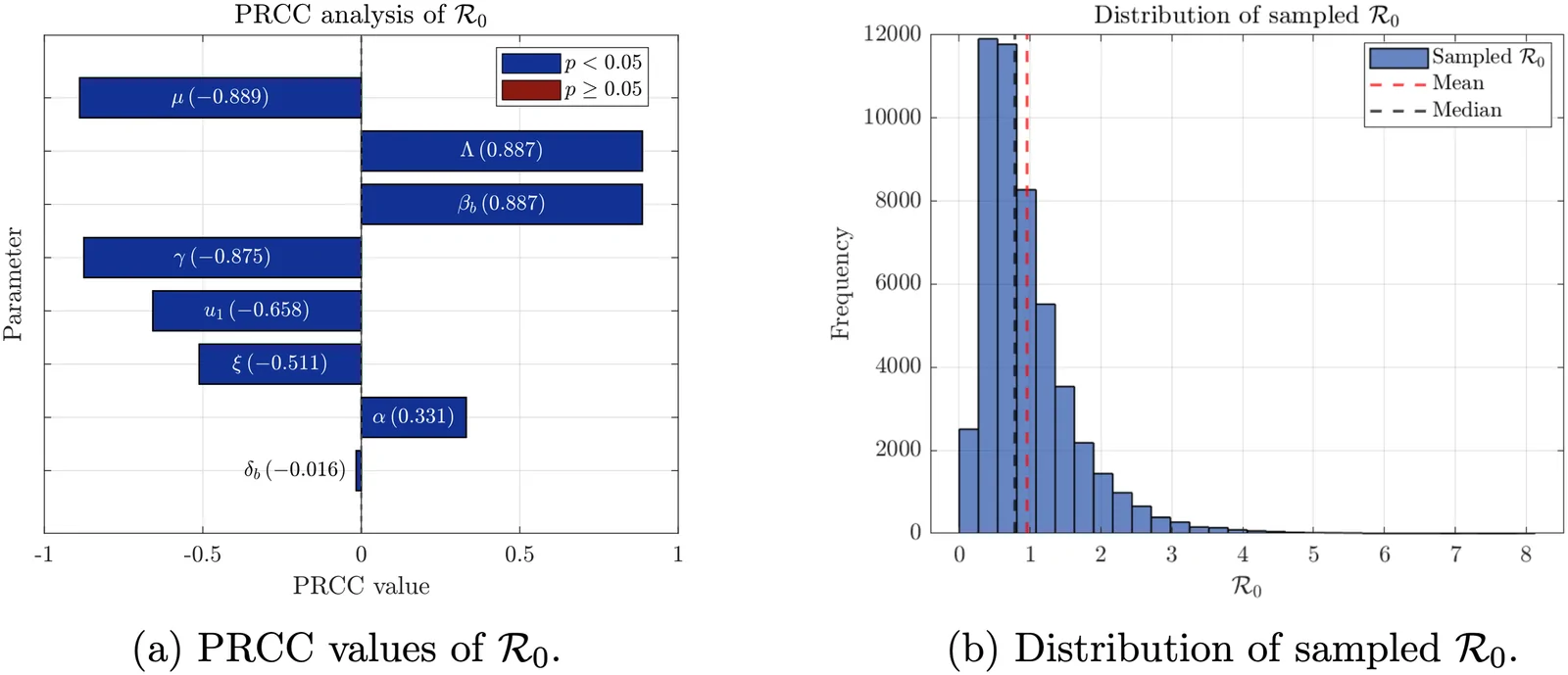
PRCC sensitivity analysis and sampled distribution of ${ℛ}_{0}$. PRCC values of the model parameters with respect to ${ℛ}_{0}$ and the distribution of the sampled ${ℛ}_{0}$ values used in the analysis.

The first sensitivity analysis is conducted for ${ℛ}_{0}$, where the results are depicted in Fig 5. In Fig 5(b), we can see the distribution of ${ℛ}_{0}$ obtained from 50000 sampling points within the feasible region. It can be seen that the mean value of ${ℛ}_{0}$ from these sampling points is 1.07. The PRCC results are depicted in Fig 5(a), where we can see that $\mu ,\Lambda ,\beta_{b},\gamma ,$ and *u*_1_ are the five most significant parameters in determining ${ℛ}_{0}$. However, among these five parameters, μ and Λ are not possible to be controlled. Hence, we ignore these parameters in the further discussion. Furthermore, we also notice that since *u*_2_ not appears in ${ℛ}_{0}$, then we will have no PRCC values for *u*_2_ on this experiment.

**Fig 6:**
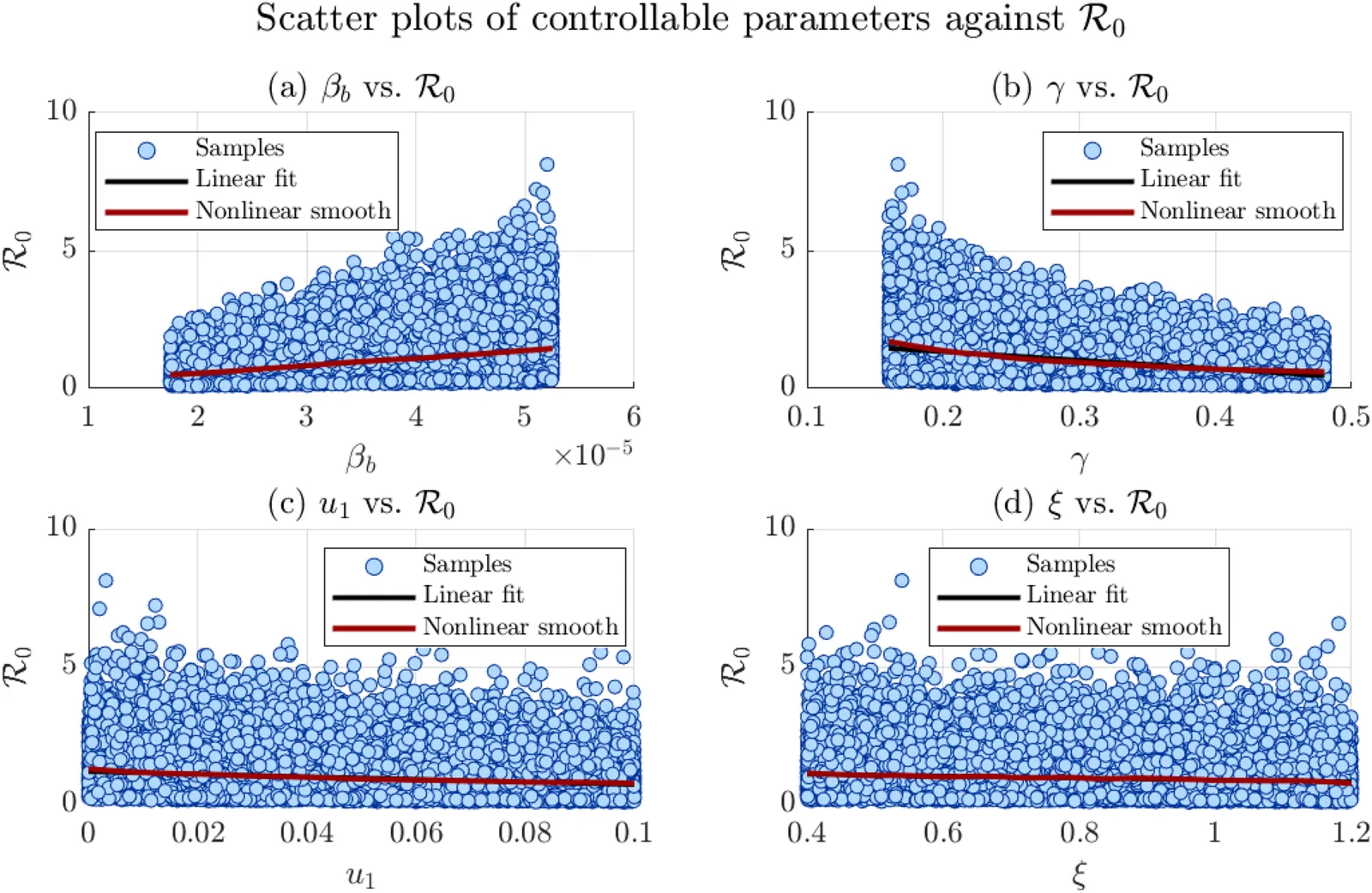
Relationships between ${ℛ}_{0}$ and selected model parameters. Scatter plots showing the relationships between ${ℛ}_{0}$ and the parameters $\beta_{b}$, γ, *u*_1_, and ξ, together with their linear and nonlinear trend fits.

We observe that $\beta_{b}$ has a positive PRCC value, indicating that increasing this parameter will increase ${ℛ}_{0}$. This result is supported by the scatter plot of $\beta_{b}$ with respect to ${ℛ}_{0}$ in Fig 6 (panel (a)). The next most significant controllable parameter is the recovery rate, denoted by γ. The PRCC value is negative, which indicates that increasing this parameter will reduce ${ℛ}_{0}$. This is also supported by the scatter plot in Fig 6 (panel (b)). For the vaccination intervention, we can see that the vaccination rate *u*_1_ and its efficacy ξ have negative PRCC values. This result means that increasing the vaccination rate as well as its efficacy will reduce ${ℛ}_{0}$. Fig 6, in panels (c) and (d), supports this result.

**Fig 7:**
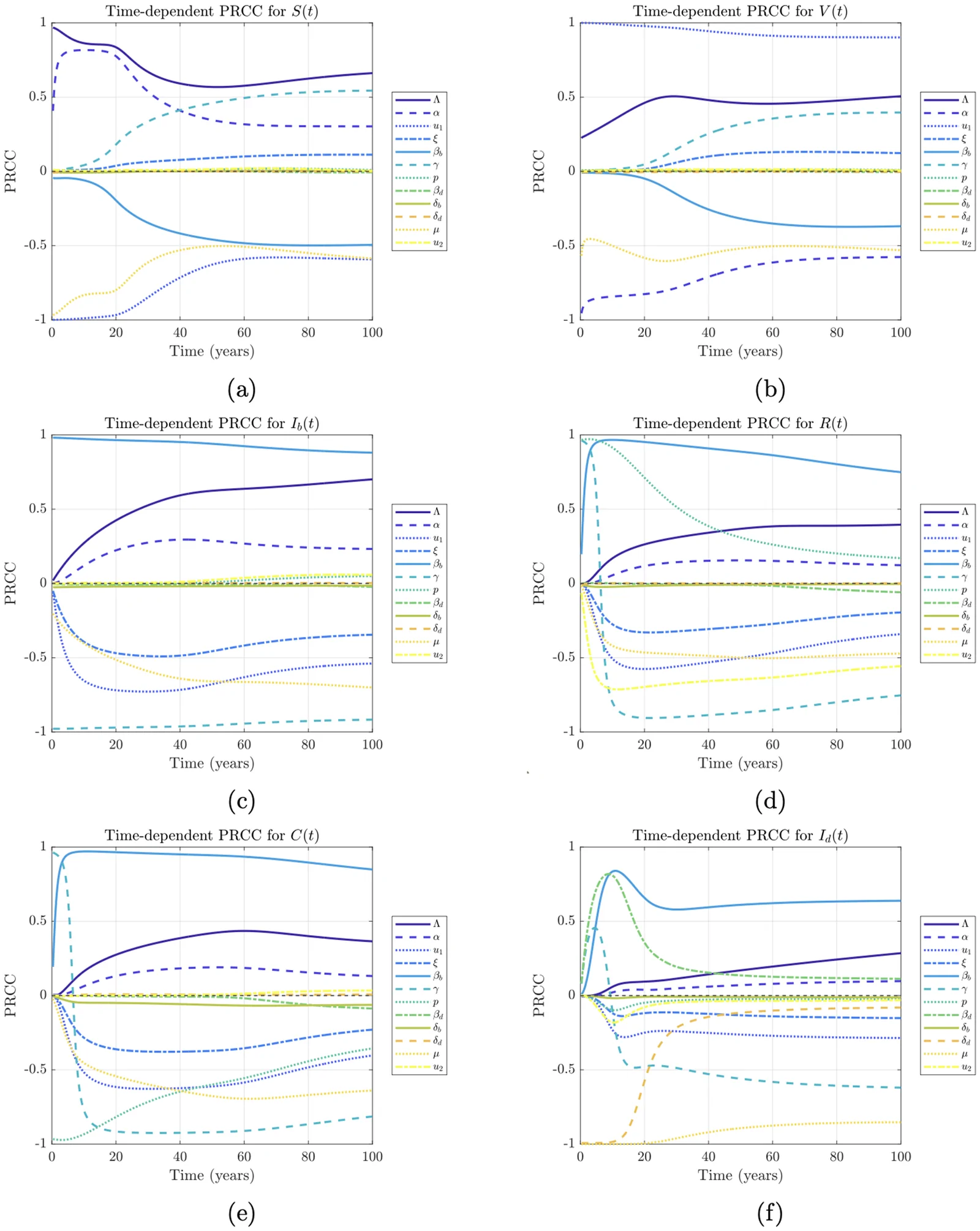
Time-dependent sensitivity analysis of the model state variables. Time-dependent PRCC values of the model parameters with respect to *S*(*t*), *V*(*t*), $I_{b}(t)$, *R*(*t*), *C*(*t*), and $I_{d}(t)$ over the simulation period.

The next global sensitivity analysis is conducted with respect to each variable in system (1), using the same parameter region as in the previous PRCC experiment for ${ℛ}_{0}$. The results are depicted in Fig 7.

The vaccination rate *u*_1_ has a positive PRCC value at *t* = 100 for the variable *V* only, and negative values for all other variables. This means that the vaccination rate successfully reduces the number of infected individuals, not only for HBV-infected individuals but also for HDV. The other intervention, namely liver transplantation, shows small positive PRCC values for $S,V,I_{b},$ and *C*. It is not difficult to understand that liver transplantation increases the number of susceptible individuals; hence, we observe positive PRCC values of *u*_2_ with respect to *S* and *V*. However, it also has positive values for $I_{b}$ and *C*, which can be explained by the fact that more successful liver transplantation increases the source “pool” for new HBV infections.

The negative PRCC values of *u*_2_ with respect to *R* and $I_{d}$ indicate that increasing the liver transplantation rate will reduce the number of individuals in *R* and decrease $I_{d}$. This is also biologically consistent, as liver transplantation provides protection against HDV infection. Another important result is observed for the impact of vaccine efficacy. It has large negative PRCC values for all infected compartments and positive PRCC values for non-infected compartments. Hence, improving vaccine efficacy has a similar level of importance as increasing the vaccination rate. A high vaccination rate combined with high vaccine efficacy will enhance the reduction of both HBV and HDV in the population.

### 4.3 Level set of the basic reproduction number and the autonomus simulations

**Fig 8:**
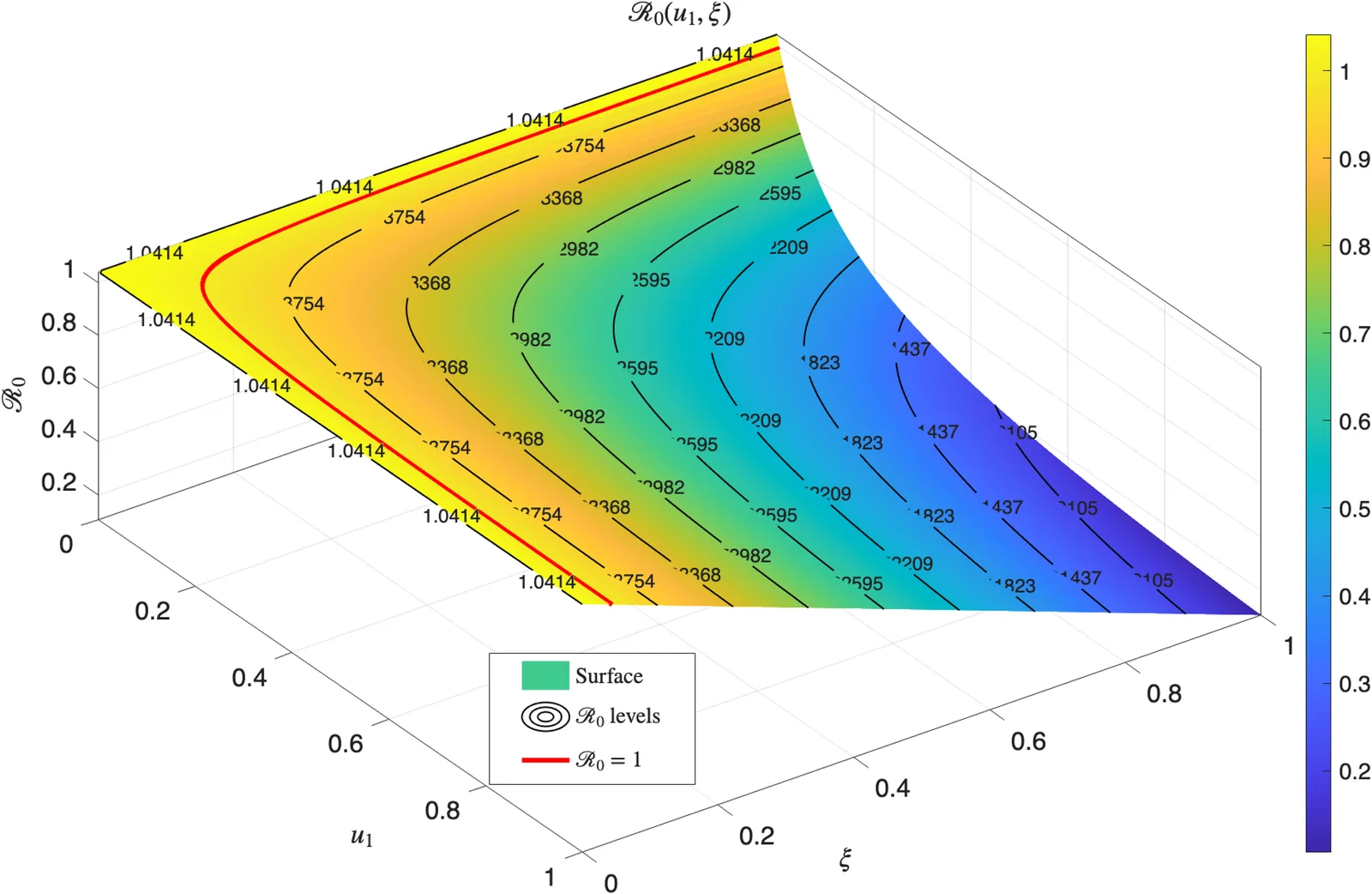
Combined effects of *u*_1_ and ξ on the basic reproduction number. Three-dimensional surface and contour plots of ${ℛ}_{0}$ as a function of *u*_1_ and ξ. The red curve indicates the threshold ${ℛ}_{0}=1$.

The next sensitivity analysis is a local sensitivity analysis conducted through the level set of ${ℛ}_{0}$, using the baseline parameter values presented in Table 2. The results are depicted in Fig 8, where we present ${ℛ}_{0}$ as a function of the vaccination rate *u*_1_ and its efficacy ξ. The red curve represents the threshold ${ℛ}_{0}=1$. It can be clearly observed that increasing the vaccination rate and/or improving vaccine efficacy will reduce ${ℛ}_{0}$.

**Fig 9:**
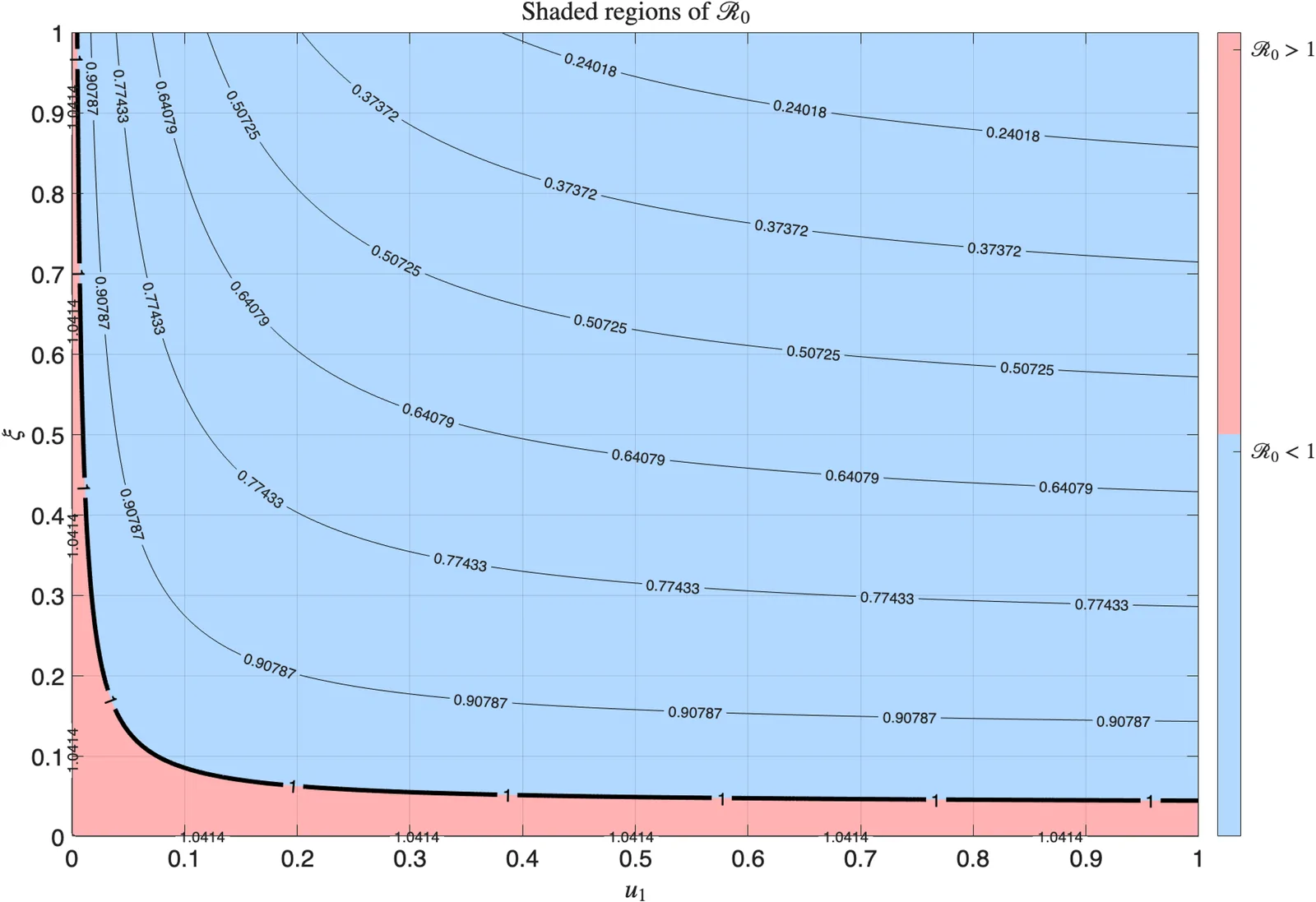
Threshold regions of ${ℛ}_{0}$ in the $\left(u_{1},\xi \right)$ parameter plane. Contour plot showing the regions where ${ℛ}_{0}<1$ and ${ℛ}_{0}>1$ as functions of *u*_1_ and ξ. The black curve represents the threshold ${ℛ}_{0}=1$ separating the two regions.

**Fig 10:**
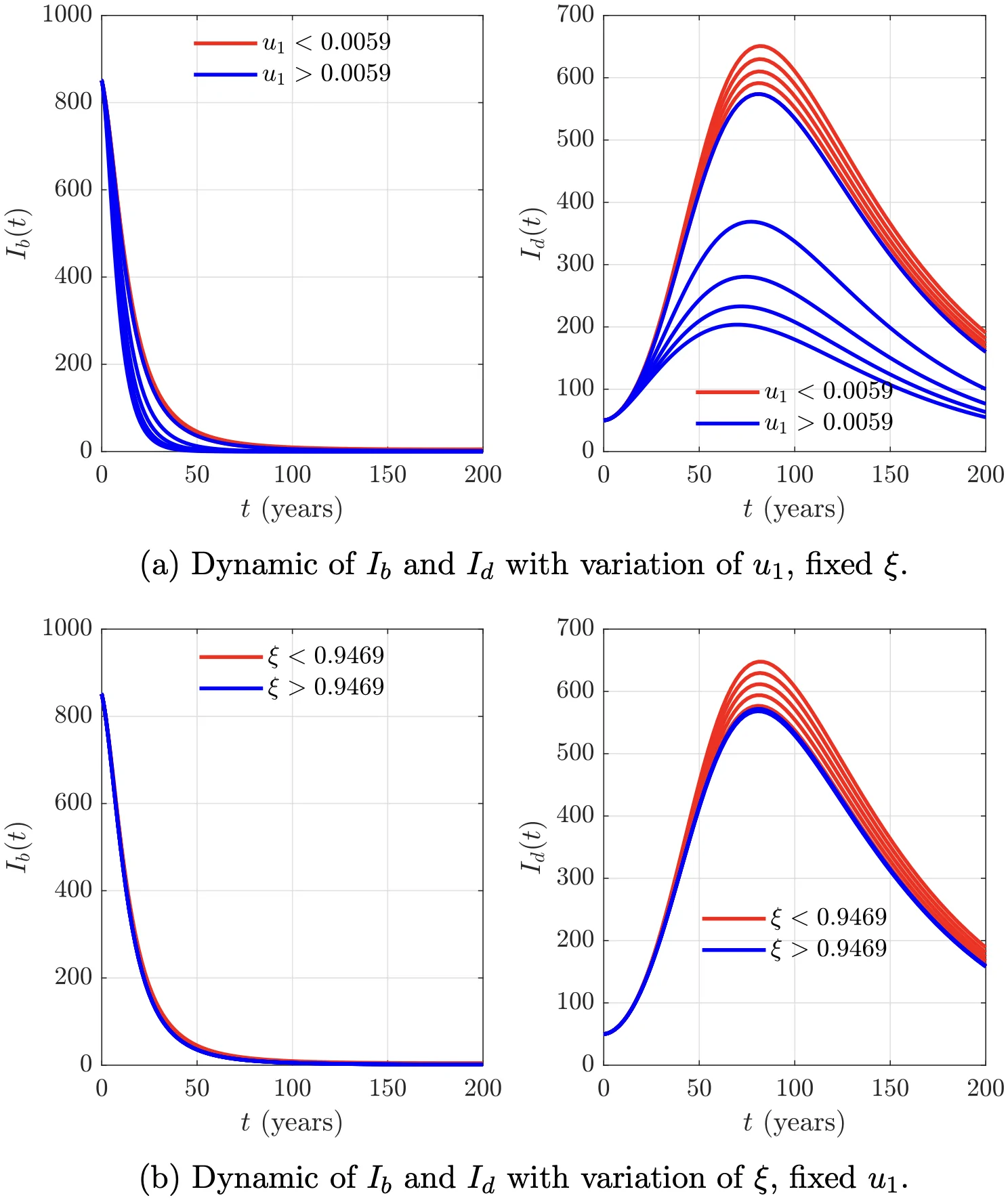
Effects of *u*_1_ and ξ on the dynamics of infected populations. Time-series dynamics of $I_{b}(t)$ and $I_{d}(t)$ under variations in *u*_1_ and ξ. The red curves correspond to parameter combinations yielding ${ℛ}_{0}>1$, whereas the blue curves correspond to combinations yielding ${ℛ}_{0}<1$.

To represent these results more clearly, we provide a shaded region of ${ℛ}_{0}$ to distinguish the epidemiological regimes as shown in Fig 9. The blue region corresponds to ${ℛ}_{0}<1$, where the disease-free equilibrium (DFE) exists and is stable, while the red region corresponds to ${ℛ}_{0}>1$, where the endemic equilibrium *EE*_1_ and *EE*_2_ exist. It can be seen that a lower vaccination rate requires a higher level of vaccine efficacy, indicated by a larger ξ. For example, if *u*_1_ is only 0.005, then a minimum vaccine efficacy of 94.69% is required to achieve effective HBV control. On the other hand, if a high-quality HBV vaccine is available, for instance with an efficacy of 80%, then a vaccination rate of only *u*_1_ = 0.0059 per year is sufficient to achieve HBV elimination. Fig 10 provides an illustration of how variations in *u*_1_ and ξ influence the dynamics of infected individuals in system (1). It can be observed that increasing the vaccination rate or improving vaccine efficacy not only reduces the magnitude of the outbreak, but also delays the timing of the outbreak peak.

**Fig 11:**
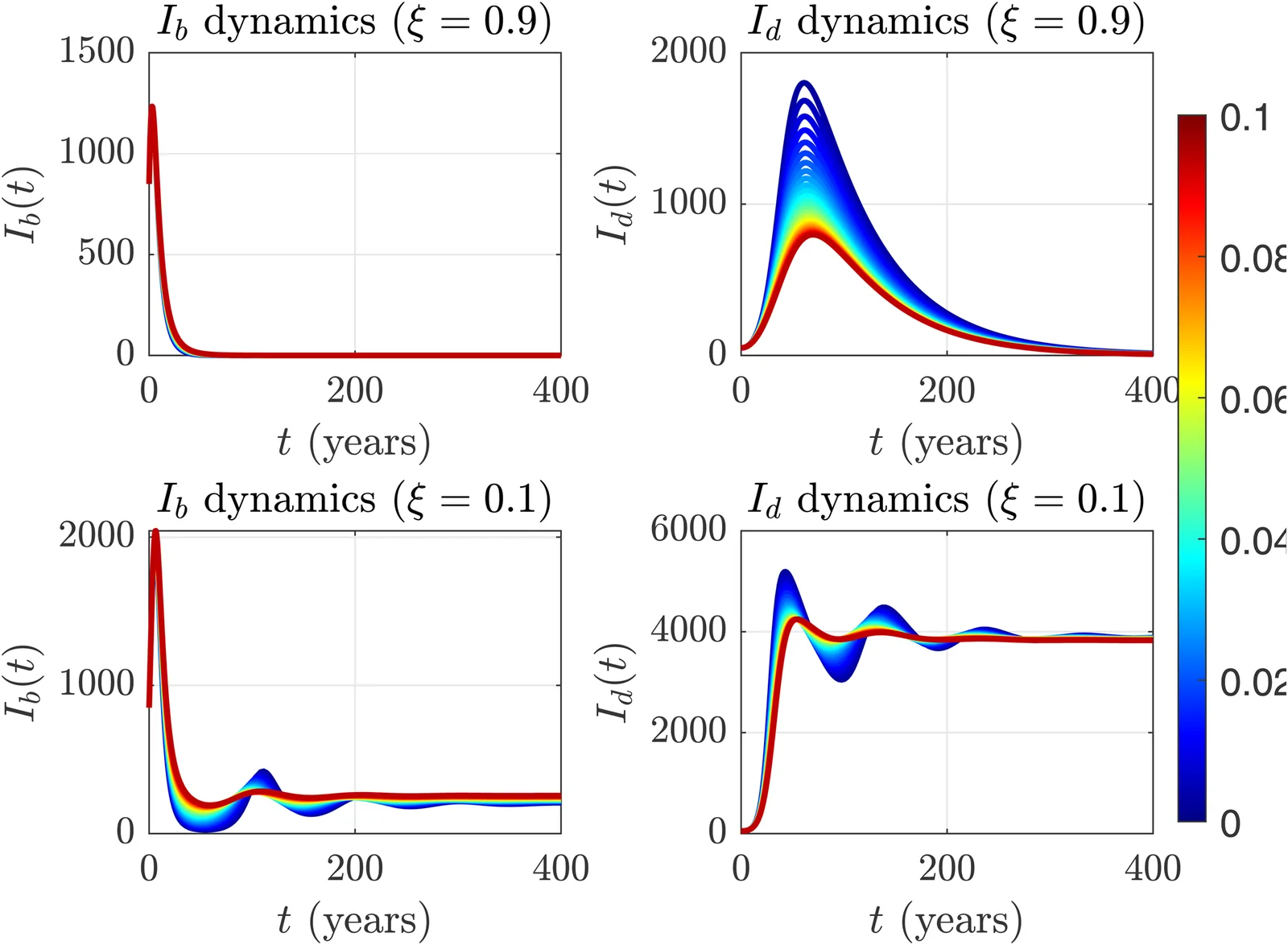
Effects of *u*_2_ and vaccine efficacy on HBV–HDV dynamics. Time-series dynamics of $I_{b}(t)$ and $I_{d}(t)$ under variations in *u*_2_ for high vaccine efficacy (ξ=0.9) and low vaccine efficacy (ξ=0.1). The colour bar indicates the values of *u*_2_ used in the simulations.

The last numerical experiment in this section is conducted to assess the impact of *u*_2_ on the dynamics of HBV and HDV. We acknowledge that although *u*_2_ does not influence the magnitude of ${ℛ}_{0}$, since it does not appear in the expression of ${ℛ}_{0}$, it has a substantial impact on the dynamics of *R* and $I_{d}$, as indicated by the PRCC results. Hence, we analyze the effect of varying *u*_2_ on the dynamics of HBV and HDV using the same parameter values as in Table 2, except that $\beta_{b}$ is set to be twice its baseline value. We also consider *u*_1_ = 0.2 with ξ=0.9, which gives ${ℛ}_{0}=0.889$, and ξ=0.1, which gives ${ℛ}_{0}=1.95$. The results are depicted in Fig 11. It can be observed that when liver transplantation intervention is accompanied by high vaccine efficacy, resulting in a smaller ${ℛ}_{0}$ (see the top panels of Fig 11), both HBV and HDV tend rapidly toward the disease-free equilibrium. In this case, the outbreak is not only reduced in magnitude but also delayed. On the other hand, when liver transplantation is combined with low vaccine efficacy (see the bottom panels of Fig 11), the intervention remains effective in reducing the outbreak amplitude and accelerates the time required to reach equilibrium.

## 5. Conclusion

In this paper, we introduced a novel transmission model of Hepatitis B virus (HBV) with superinfection by Hepatitis D virus (HDV). It is assumed that co-infection is not possible, and that HDV transmission occurs only through superinfection in individuals who are already infected with HBV. Vaccination and liver transplantation are incorporated into the model as potential interventions to reduce disease transmission. The model is formulated as a system of six-dimensional ordinary differential equations, where the total human population is divided into susceptible, vaccinated, HBV-infected, recovered, chronic, and HDV-infected compartments.

Mathematical analysis regarding the existence and stability of the equilibrium points is conducted rigorously. We find that the disease-free equilibrium is always locally asymptotically stable when the basic reproduction number is less than one, and unstable when it exceeds one. The model also admits endemic equilibrium, which can be classified into two types. The first type corresponds to the absence of HDV infection, while the second type represents the coexistence of HBV and HDV infection. Both types of endemic equilibrium exist whenever ${ℛ}_{0}>1$. From the continuation analysis using *MatCont*, we identify two branching points that determine the stability regions of each equilibrium. The first branching point occurs at ${ℛ}_{0}=1$, which separates the stable and unstable regions of the disease-free equilibrium and marks the emergence of endemic equilibria. The second branching point, which occurs at a value larger than ${ℛ}_{0}=1$, defines a critical threshold governing the stability of the two endemic equilibria. When ${ℛ}_{0}$ lies between these two branching points, the endemic equilibrium without HDV is stable, while the endemic equilibrium with HDV is unstable. Beyond the second branching point, the endemic equilibrium without HDV loses its stability, and the endemic equilibrium with HDV becomes stable.

A global sensitivity analysis using Partial Rank Correlation Coefficient (PRCC) combined with Latin Hypercube Sampling (LHS) is conducted to assess the impact of parameter values on ${ℛ}_{0}$ and the dynamics of each variable in the proposed model. We find that higher vaccination coverage and better vaccine efficacy can significantly reduce the magnitude of the basic reproduction number. Furthermore, although liver transplantation does not affect the value of ${ℛ}_{0}$, it has a notable impact on the disease dynamics. We show that a higher level of liver transplantation not only reduces the magnitude of HBV and HDV outbreaks, but also delays the timing of the outbreak peak.

Overall, these findings highlight the importance of combining preventive and clinical interventions in controlling HBV–HDV transmission. Since HDV infection in the present model can arise only after HBV infection, improving HBV vaccination coverage and vaccine efficacy can indirectly reduce the population at risk of HDV superinfection. Vaccination therefore remains the primary strategy for reducing transmission potential, whereas liver transplantation plays a complementary role by reducing outbreak magnitude and delaying outbreak peaks without directly changing ${ℛ}_{0}$. This suggests that relying solely on ${ℛ}_{0}$ may underestimate the role of treatment-based interventions in shaping epidemic outcomes. Therefore, an integrated public health strategy that combines high vaccination coverage with effective clinical management is essential to achieve sustainable control and potential elimination of HBV and HDV infection.

Future work may extend the present framework by incorporating simultaneous HBV–HDV coinfection, vertical HBV transmission, and more detailed clinical stages of HDV infection. It would also be valuable to include HDV-specific treatment responses and disease progression toward severe liver complications. In addition, combining the model with parameter estimation from real epidemiological data and an optimal-control framework could help identify cost-effective strategies for allocating vaccination, treatment, and liver-transplantation resources. Future work may also consider a stochastic extension of the present HBV–HDV framework to capture random variability in transmission, treatment outcomes, and disease progression, which may be particularly relevant for small populations and early outbreak dynamics [60–62].

## Supporting information

S1 File Expression of polynomial for existence of *EE*_2_. (PDF)

S2 FileExpression of $\beta_{d}^{*}$.(PDF)
