# Supplementary material for: Mathematical modeling of HBV–HDV transmission with superinfection: The role of vaccination and liver transplantation in disease dynamics
Source: PLoS One. 2026 Sep 9;21(9):e0356725. doi: 10.1371/journal.pone.0356725 (PMC13557504; doi:10.1371/journal.pone.0356725)
Supplement: Supplementary file 1 [file pone.0356725.s001.pdf]

S1 Appendix: Expression of polynomial for existence of EE_2

The existence of EE_2 depends on the positive roots of the following polynomial:

$$a_4 I_d^4 + a_3 I_d^3 + a_2 I_d^2 + a_1 I_d + a_0 = 0$$

where

$$a_4 = (\xi - 1)\beta_d^4\Big(\mu^2 + (\delta_b + \delta_d + \gamma)\mu + \delta_d\gamma + \delta_b\delta_d\Big)(\mu + \delta_d)\beta_b^2,$$

$$\begin{aligned}
a_3 = -\beta_b\Bigg[&(\xi - 1)\Big(- 4\mu^3 + (-6\delta_b - 4\delta_d - 4\gamma - 2u_2)\mu^2 \\
&+ \big((-4\delta_d - 2\delta_b + (p-2)u_2)\gamma - 2\delta_b^2 + (-6\delta_d - 2u_2)\delta_b - 2\delta_d u_2\big)\mu \\
&+ \big((-2\delta_b\delta_d + (p-2)u_2\delta_d + \beta_d\Lambda)\gamma - 2\delta_b\delta_d(\delta_b + u_2)\big)\Big)\beta_b \\
&- \Big((\xi - 2)\mu + \xi u_1 - \alpha - u_1\Big)\beta_d\gamma(\gamma + \delta_b + \mu)\Bigg](\mu + \delta_d)\beta_d^3,
\end{aligned}$$

$$\begin{aligned}
a_2 = \Bigg(&(\mu + \delta_d)(1 - \xi)\Bigg[- 6\mu^4 + 6(-2\delta_b - \delta_d - \gamma - u_2)\mu^3 \\
&+ \Big(3((p-2)u_2 - 2\delta_b - 2\delta_d)\gamma + 6(-2\delta_b - u_2)\delta_d - 7\delta_b^2 - 10\delta_b u_2 - u_2^2\Big)\mu^2 \\
&+ \Big((3\beta_d\Lambda + 3((p-2)u_2 - 2\delta_b)\delta_d - \delta_b^2 + 2(p-2)u_2\delta_b + u_2^2(p-1))\gamma \\
&\quad + (-7\delta_b^2 - 10\delta_b u_2 - u_2^2)\delta_d - \delta_b^3 - 4u_2\delta_b^2 - u_2^2\delta_b\Big)\mu \\
&+ \Big(\Lambda((p+1)\delta_b - (p-2)u_2)\beta_d \\
&\quad + 2\delta_d\Big(- \tfrac{1}{2}\delta_b^2 + (p-2)u_2\delta_b + \tfrac{1}{2}u_2^2(p-1)\Big)\Big)\gamma \\
&- \delta_d\delta_b(\delta_b^2 + 4\delta_b u_2 + u_2^2)\Bigg]\beta_b^2 \\
&+ \beta_d\gamma\Bigg[3(\xi - 2)\mu^4 \\
&+ \Big(3(\xi - 2)\gamma + 3(\xi - 2)\delta_d + (\xi - 2)(p+4)\delta_b - (\xi - 2)(p-2)u_2 + 3\xi u_1 - 3\alpha - 3u_1\Big)\mu^3 \\
&+ \Big((3(\xi - 2)\delta_d + (\xi - 2)(p+1)\delta_b + ((-p+2)\xi + 3p - 4)u_2 + 3\xi u_1 - 3\alpha - 3u_1)\gamma \\
&\quad + ((\xi - 2)(p+4)\delta_b - (\xi - 2)(p-2)u_2 + 3\xi u_1 - 3\alpha - 3u_1)\delta_d \\
&\quad + (\xi - 2)(p+1)\delta_b^2 + (-(\xi - 2)(p-2)u_2 + (\xi u_1 - \alpha - u_1)(p+4))\delta_b \\
&\quad - u_2(\xi u_1 - \alpha - u_1)(p-2)\Big)\mu^2 \\
&+ \Big((\beta_d\Lambda + ((\xi - 2)(p+1)\delta_b + ((-p+2)\xi + 3p - 4)u_2 + 3\xi u_1 - 3\alpha - 3u_1)\delta_d \\
&\quad + (\xi u_1 - \alpha - u_1)((p+1)\delta_b - 2u_2(p-1)))\gamma \\
&\quad + ((\xi - 2)(p+1)\delta_b^2 + (-(\xi - 2)(p-2)u_2 + (\xi u_1 - \alpha - u_1)(p+4))\delta_b
\end{aligned}$$

$$
\begin{aligned}
&\quad - u_2(\xi u_1 - \alpha - u_1)(p-2))\delta_d \\
&\quad + (\xi u_1 - \alpha - u_1)((p+1)\delta_b - (p-2)u_2)\delta_b\Big)\mu \\
&+ (\xi u_1 - \alpha - u_1)\Big[(-\beta_d\Lambda + \delta_d((p+1)\delta_b - 2u_2(p-1)))\gamma \\
&\quad + \delta_d((p+1)\delta_b - (p-2)u_2)\delta_b\Big]\Bigg]\beta_b \\
&- \gamma^2(\gamma + \delta_b + \mu)(\alpha + \mu + u_1)\mu\beta_d^2\Bigg)\beta_d^2.
\end{aligned}
$$

$$
\begin{aligned}
a_1 = (1-\xi)(\mu+\delta_d)(\mu+\delta_b)\Bigg[&(\mu+\delta_b)\Big(\Lambda\gamma(p\delta_b - \mu u_2 + \mu + u_2)\beta_d \\
&+ (\mu+\delta_d)(\mu+\delta_b)\big(-\mu^2 + (-\delta_b - \gamma - u_2)\mu + u_2((p-1)\gamma - \delta_b)\big)\Big)\beta_d \\
&+ \Bigg[(\mu+\delta_b)\Bigg(\Lambda\gamma\beta_d^2 + \Big(-2\mu^3 - 3\big(\delta_b + \tfrac{2\delta_d}{3} + \tfrac{2\gamma}{3} + \tfrac{u_2}{3}\big)\mu^2 \\
&\quad + \big((pu_2 - \delta_b - 2\delta_d - u_2)\gamma - (\delta_b+\delta_d)u_2 - \delta_b(\delta_b + 3\delta_d)\big)\mu \\
&\quad + \delta_d(pu_2 - \delta_b - u_2)\gamma - \delta_b\delta_d(\delta_b + u_2)\Big)\beta_d\Bigg) \\
&+ \beta_d\Bigg(\Lambda\gamma(p\delta_b - pu_2 + \mu + u_2)\beta_d \\
&+ (\mu+\delta_d)(\mu+\delta_b)\big(-\mu^2 + (-\delta_b - \gamma - u_2)\mu + u_2((p-1)\gamma - \delta_b)\big)\Bigg)\Bigg](\mu+u_2)\beta_b^2 \\
&- \beta_d^2\beta_b\Bigg[-(\xi-1)\Bigg(p(\mu+\delta_d)\delta_b^3 + 3\left(\frac{2u_2}{3} + (\mu + \frac{\gamma}{3})p + \frac{2\mu}{3}\right)(\mu+\delta_d)\delta_b^2 \\
&+ \Big(-(\mu+\delta_d)(p-1)u_2^2 - (p^2\gamma + (2\mu+\gamma)p - 6\mu - 2\gamma)(\mu+\delta_d)u_2\Big) \\
&+ 2((\mu+\gamma)p + \tfrac{5\mu}{2} + \gamma)\mu\delta_d + (-2\Lambda\gamma\beta_d + 2\gamma\mu^2 + 2\mu^3)p + 5\mu^3 + 2\gamma\mu^2\Big)\delta_b \\
&+ (\mu+\delta_d)(p-1)(\gamma p - \gamma - \mu)u_2^2 \\
&+ \Big(-2\mu((\mu+2\gamma)p - 2\mu - 2\gamma)\delta_d \\
&+ (2\Lambda\gamma\beta_d - 4\gamma\mu^2 - 2\mu^3)p + 4\mu^3 + 4\gamma\mu^2 - 2\Lambda\gamma\beta_d\Big)u_2 \\
&+ 3\mu\big(\mu(\mu+\gamma)\delta_d + \mu^3 + \gamma\mu^2 - \tfrac{2}{3}\Lambda\gamma\beta_d)\Bigg)u_1 \\
&+ 2(\gamma + \delta_b + \mu)\Big(-\tfrac{1}{2}p\delta_B^2 + (-\mu p - \mu - u_2)\delta_b \\
&+ u_2^2\left(\tfrac{p}{2} - \tfrac{1}{2}\right) + \mu(p-2)u_2 - \tfrac{3}{2}\mu^2\Big) \\
&+ \mu(\mu+\delta_d)\xi + 2p\left(\mu + \tfrac{\alpha}{2}\right)(\mu+\delta_d)\delta_b^3 \\
&+ 6\left(\frac{2u_2}{3} + \left(\mu + \frac{\gamma}{3}\right)p + \frac{2\mu}{3}\right)\left(\mu + \frac{\alpha}{2}\right)(\mu+\delta_d)\delta_b^2 \\
&+ \left(-2(p-1)\left(\mu + \frac{\alpha}{2}\right)(\mu+\delta_d)\right)u_2^2 \\
&- \left((p^2\gamma + (2\mu+\gamma)p - 6\mu - 2\gamma)\alpha + \mu(p^2\gamma + (4\mu+\gamma)p - 12\mu - 4\gamma)\right)(\mu+\delta_d)u_2 \\
&+ 4((\mu+\gamma)p + \tfrac{5\mu}{2} + \gamma)\mu(\mu + \tfrac{\alpha}{2})\delta_d + \big((-2\Lambda\gamma\beta_d + 2\gamma\mu^2 + 2\mu^3)p + 5\mu^3 + 2\gamma\mu^2\big)\alpha
\end{aligned}
$$

$$+ 4\big((\mu^3 + \gamma\mu^2 - \tfrac{1}{2}\Lambda\gamma\beta_d)p + (\tfrac{5\mu^3}{2} + \gamma\mu^2)\mu\big)\delta_b + \big((\gamma p - \gamma - \mu)\alpha +$$

$$\mu(\gamma p - 2\gamma - 2\mu)\big)(p-1)(\mu + \delta_d)u_2^2 + \big(- 4\mu\left(\left(\tfrac{\mu}{2} + \gamma\right)p - \mu - \gamma\right)\alpha$$

$$+ \mu\left((\mu + \tfrac{3\gamma}{2})p - 2\mu - 2\gamma\right)\delta_d + \big((2\Lambda\gamma\beta_d - 4\gamma\mu^2 - 2\mu^3)p + 4\mu^3 + 4\gamma\mu^2 - 2\Lambda\gamma\beta_d\big)\alpha$$

$$- 4((\mu^3 + \tfrac{3}{2}\gamma\mu^2 - \tfrac{1}{2}\Lambda\gamma\beta_d)p - 2\mu^3 - 2\gamma\mu^2 + \tfrac{1}{2}\Lambda\gamma\beta_d)\mu\big)u_2$$

$$+ 6\mu\big((\mu+\gamma)\mu(\mu + \tfrac{\alpha}{2})\delta_d + (\tfrac{1}{2}\mu^3 + \tfrac{1}{2}\gamma\mu^2 - \tfrac{1}{3}\Lambda\gamma\beta_d)\alpha + \mu(\mu^3 + \gamma\mu^2 - \tfrac{1}{3}\Lambda\gamma\beta_d)\big)\Big)\gamma$$

$$- 2\gamma^2(\gamma + \delta_b + \mu)(\alpha + \mu + u_1)\mu\beta_d^3(p\delta_b - pu_2 + \mu + u_2)\Bigg],$$

$$a_0 = (1-\xi)(\mu + u_2)\Bigg[\Lambda\gamma(p\delta_b - \mu u_2 + \mu + u_2)\beta_d$$

$$+ (\mu + \delta_d)(\mu + \delta_b)\big(- \mu^2 + (-\delta_b - \gamma - u_2)\mu + u_2((p-1)\gamma - \delta_b)\big)\Bigg](\mu + \delta_b)(\mu + \delta_d)\beta_b^2$$

$$+ \gamma\Bigg[\Lambda\gamma\big(\mu^2 + (p\delta_b - \mu u_2 - \xi u_1 + \alpha + u_1 + u_2)\mu$$

$$- (\xi u_1 - \alpha - u_1)(p\delta_b - \mu u_2 + u_2)\big)\beta_d$$

$$+ \Big((\xi - 2)\mu^3 + ((\xi-2)(\gamma + u_2 + \delta_b) + \xi u_1 - \alpha - u_1)\mu^2$$

$$+ ((pu_2 + \xi u_1 + \xi u_2 - \alpha - u_1 - 2u_2)\gamma - (-\xi\delta_b - \xi u_1 + \alpha + 2\delta_b + u_1)u_2$$

$$- \delta_b(-\xi u_1 + \alpha + u_1))\mu$$

$$- u_2((p-1)\gamma - \delta_b)(\xi u_1 - \alpha - u_1)\Big)(\mu + \delta_d)(\mu + \delta_b)\Bigg]\beta_d$$

$$- \gamma^2(\gamma + \delta_b + \mu)(\alpha + \mu + u_1)\mu\beta_d^2(p\delta_b - \mu u_2 + \mu + u_2)^2.$$
