# Supplementary material for: Mathematical modeling of HBV–HDV transmission with superinfection: The role of vaccination and liver transplantation in disease dynamics
Source: PLoS One. 2026 Sep 9;21(9):e0356725. doi: 10.1371/journal.pone.0356725 (PMC13557504; doi:10.1371/journal.pone.0356725)
Supplement: Supplementary file 2 [file pone.0356725.s002.pdf]

S2 Appendix: Expression of β_d^*

$$
\begin{aligned}
\beta_d^* = \Bigg(\beta_b\Bigg[&-(\mu+\delta_b)(\mu+\delta_d)\Big(-\mu^3+(-\delta_b-\gamma-u_1-u_2)\mu^2\\
&+\Big((-\delta_b-\gamma-u_2)u_1+(-\gamma-\delta_b)u_2+\Lambda\beta_b\Big)\mu\\
&+u_2\big((\gamma p-\gamma-\delta_b)u_1+\Lambda\beta_b\big)\Big)\xi\\
&+2\mu^3+(2\delta_b+\alpha+2\gamma+u_1+2u_2)\mu^2\\
&+\big((\delta_b+\gamma+u_2)u_1+(-\gamma p+\alpha+2\gamma+2\delta_b)u_2\\
&+\alpha(\delta_b+\gamma)-\Lambda\beta_b\big)\mu\\
&-u_2(\gamma p-\gamma-\delta_b)u_1+\big((-\gamma p+\gamma+\delta_b)\alpha-\Lambda\beta_b\big)u_2\\
&+\Big((\mu+u_2)^2\Big(\alpha^2+((-2\xi+2)u_1+2\mu\xi)\alpha+((-1+\xi)u_1+\mu\xi)^2\Big)\delta_b^2\\
&-2(\mu+u_2)\Big[(pu_2-\mu-u_2)\alpha^2+\big(u_2((-2\xi+2)u_1+\mu(\xi+1))p\\
&-2((-\xi+1)u_1+\mu\xi)(\mu+u_2)\big)\alpha\\
&+u_2\big((-1+\xi)^2u_1^2+\mu(-1+\xi)^2u_1+\mu^2\xi\big)p\\
&-(\mu+u_2)\big((-1+\xi)u_1+\mu\xi\big)^2)\gamma\\
&-(\mu+u_2)\Big[\alpha^2\mu+(-2\mu(-1+\xi)u_1-\Lambda\beta_b(-1+\xi)+2\mu^2\xi)\alpha\\
&\quad+\mu(-1+\xi)^2u_1^2+2(-1+\xi)\big(\Lambda\beta_b(-1+\xi)/2+\mu^2\xi\big)u_1\\
&\quad+\xi(-\Lambda\beta_b(-1+\xi)+\mu^2\xi)\mu\Big]\delta_b\\
&+(pu_2-\mu-u_2)^2\alpha^2\\
&+\Big[2\big(((-\xi+1)u_1+\mu)u_2p-((-\xi+1)u_1+\mu\xi)(\mu+u_2)\big)(pu_2-\mu-u_2)\alpha\\
&+\big((-\xi+1)u_1+\mu\big)^2u_2^2p^2-2u_2(\mu+u_2)\big((-1+\xi)^2u_1^2+\mu(-1+\xi)^2u_1+\mu^2\xi\big)p\\
&+(\mu+u_2)^2\big((-1+\xi)u_1+\mu\xi\big)^2\Big]\gamma^2\\
&-2(\mu+u_2)\Big[\mu(pu_2-\mu-u_2)\alpha^2+\Big(u_2\big(-2\mu(-1+\xi)u_1-\Lambda\beta_b(-1+\xi)+\mu^2(\xi+1)\big)p\\
&\quad-2(\mu+u_2)(-\mu(-1+\xi)u_1-\Lambda\beta_b(-1+\xi)/2+\mu^2\xi)\Big)\alpha\\
&+u_2\Big(\mu(-1+\xi)^2u_1^2+(-1+\xi)^2(\Lambda\beta_b+\mu^2)u_1\\
&\quad+(-\Lambda\beta_b(-1+\xi)+\mu^2\xi)\mu\Big)p\\
&-(\mu+u_2)(\mu(-1+\xi)^2u_1^2)\\
&+2(-1+\xi)\Big(\Lambda\beta_b(-1+\xi)/2+\mu^2\xi\Big)u_1-\xi(-\Lambda\beta_b(-1+\xi)+\mu^2\xi)\mu\Big]\gamma\\
&+(\mu+u_2)^2\Big[\alpha^2\mu^2+2(-\mu(-1+\xi)u_1-\Lambda\beta_b(-1+\xi)+\mu^2\xi)\mu\alpha\\
&\quad+\mu^2(-1+\xi)^2u_1^2+2(\Lambda\beta_b(-1+\xi)+\mu^2\xi)(-1+\xi)\mu u_1
\end{aligned}
$$

$$
+ (-\Lambda\beta_b(-1+\xi) + \mu^2\xi)^2\Big]\Big)^{1/2}\Big]\Big)
$$

$$
\Big/\Big(2(\mu + (-p+1)u_2 + p\delta_b)\gamma\big[u_1\Lambda\beta_b\xi + (\alpha+\mu+u_1)(\mu^2 + (\delta_b+\gamma)\mu - \Lambda\beta_b)\big]\Big).
$$
